# QTL for seed shattering and threshability in intermediate wheatgrass align closely with well‐studied orthologs from wheat, barley, and rice

**DOI:** 10.1002/tpg2.20145

**Published:** 2021-10-09

**Authors:** Kayla R. Altendorf, Lee R. DeHaan, Steve R. Larson, James A. Anderson

**Affiliations:** ^1^ USDA–ARS Forage Seed and Cereal Research Unit Prosser WA 99350 USA; ^2^ The Land Institute Salina KS 67401 USA; ^3^ USDA–ARS Forage & Range Research Lab Logan UT 84322 USA; ^4^ Dep. of Agronomy and Plant Genetics Univ. of Minnesota St. Paul MN 55108 USA

## Abstract

Perennial grain crops have the potential to improve agricultural sustainability but few existing species produce sufficient grain yield to be economically viable. The outcrossing, allohexaploid, and perennial forage species intermediate wheatgrass (IWG) [*Thinopyrum intermedium* (Host) Barkworth & D. R. Dewey] has shown promise in undergoing direct domestication as a perennial grain crop using phenotypic and genomic selection. However, decades of selection will be required to achieve yields on par with annual small‐grain crops. Marker‐aided selection could accelerate progress if important genomic regions associated with domestication were identified. Here we use the IWG nested association mapping (NAM) population, with 1,168 F_1_ progeny across 10 families to dissect the genetic control of brittle rachis, floret shattering, and threshability. We used a genome‐wide association study (GWAS) with 8,003 single nucleotide polymorphism (SNP) markers and linkage mapping—both within‐family and combined across families—with a robust phenotypic dataset collected from four unique year‐by‐location combinations. A total of 29 quantitative trait loci (QTL) using GWAS and 20 using the combined linkage analysis were detected, and most large‐effect QTL were in common across the two analysis methods. We reveal that the genetic control of these traits in IWG is complex, with significant QTL across multiple chromosomes, sometimes within and across homoeologous groups and effects that vary depending on the family. In some cases, these QTL align within 216 bp to 31 Mbp of BLAST hits for known domestication genes in related species and may serve as precise targets of selection and directions for further study to advance the domestication of IWG.

## Core Ideas

- The two forms of shattering in intermediate wheatgrass are under independent genetic control.
- A QTL for brittle rachis type shattering was within 216 bp of an ortholog of *Btr2*.
- A floret shattering QTL on chromosome 11 is likely novel to intermediate wheatgrass.
- Similar to wheat, threshability QTL were found primarily on Group 2 chromosomes.
- Previous research on annual cereals has informed the domestication of intermediate wheatgrass.

**Abbreviations**
GWAS: genome‐wide association study
IWG: intermediate wheatgrass
LOD: logarithm of the odds
NAM: nested association mapping
QTL: quantitative trait loci
SNP: single nucleotide polymorphism
STP: University of Minnesota Agricultural Experiment Station in St. Paul, MN
TLI: The Land Institute.

## 1 INTRODUCTION

Perennial cropping systems have the potential to provide extensive environmental service benefits compared with annual cropping systems (Glover et al., 2010; Pimentel et al., 2012). The challenge is that ∼69% of the world's agricultural land is dedicated to the production of annual crops, including cereal grains, legumes, and oilseeds (Cox et al., 2006). There are few viable perennial alternatives for these commodities, as herbaceous perennials were not domesticated during the first agricultural revolution (Van Tassel et al., 2010). Today, plant breeders are relying on focused and conscious artificial phenotype‐ and genotype‐based selection to develop crops to fill this void (DeHaan et al., 2004, 2014; Van Tassel et al., 2010). There are two distinct approaches for developing new perennial crops. The first is interspecific hybridization, in which a cultivated annual species is crossed to a related perennial and attempts are made to introgress perenniality into annuals or desirable traits into perennials. The second is direct domestication, where selection for domestication traits takes place within an undomesticated perennial species (Cox et al., 2006; Kantar et al., 2016). In the case of direct domestication, much emphasis is placed on fixing domestication syndrome traits, which are the major traits that distinguish most cultivated species from their wild progenitors (Harlan et al., 1973). These broadly include characteristics associated with improved establishment, such as lower dormancy and larger seed mass, and increased harvest efficiency through reduced shattering and improved threshability (i.e., ease of removal of the caryopsis from the lemma and palea) (Purugganan & Fuller, 2009).

One species currently undergoing direct domestication that has generated considerable interest is perennial grass species intermediate wheatgrass (IWG) [*Thinopyrum intermedium* (Host) Barkworth & D. R. Dewey] (2*n* = 6*x* = 42). Like wheat (*Triticum aestivum* L.), IWG is an allohexaploid, which evolved from the hybridization of three different species each with seven homologous chromosome pairs for each of the three homeologous chromosomes (DeHaan et al., 2020). Grain from improved lines of IWG is being marketed under the name Kernza, a trademark owned by The Land Institute (DeHaan & Ismail, 2017). Intermediate wheatgrass is a cool‐season, rhizomatous, outcrossing and largely self‐incompatible grass native to Europe and Asia (Dewey, 1962). It was introduced to North America in 1932 and has since been used extensively as a hay and pasture grass (Ogle et al., 2011). In 1988, the Rodale Institute (Kutztown, PA) selected IWG from a panel of nearly 100 perennial grasses for its domestication potential (Wagoner, 1990). Since the initial selections, several breeding programs have been established and use both phenotype‐ and genotype‐based recurrent selection methodologies (Cox et al., 2010; DeHaan et al., 2014; Zhang et al., 2016).

In contrast to ancient plant domestication events, modern‐day efforts are supported by an extensive body of literature generated over the last century that has illuminated where, why, and how annual crops cereals like barley (*Hordeum vulgare* L.), maize (*Zea mays* L.), rice (*Oryza sativa* L.), and wheat were domesticated. Plant domestication has been studied in detail within the context of our understanding of human history, artificial and natural selection, the coevolution of humans and plants, and genomics. Most relevant in this case, however, is the applicability of this knowledge to new breeding efforts (Doebley et al., 2006; Pourkheirandish et al., 2020). These resources are especially advantageous in the case of IWG, as it is a member of the Poaceae family and Triticeae tribe where gene order and content among species are highly conserved (Moore & Moser, 1995), a phenomenon recently demonstrated specifically between IWG, barley, and *Aegilops tauschii* Coss. (Kantarski et al., 2017; Zhang et al., 2016). In addition to what can be considered a blueprint for plant domestication, breeders also have access to affordable DNA sequencing, methods for trait discovery, high‐throughput phenotyping methodology, and proven methods for selection. Selection efforts thus far in the development of IWG have focused on improving grain yield on a per‐spike basis and seed size (DeHaan et al., 2018). However, many other domestication and improvement traits need attention including shattering resistance, threshability, reduced height, increased seed size, uniform flowering time, and reduced tillering (DeHaan et al., 2018; Zhang et al., 2017; Bajgain et al., 2019a; Larson et al., 2019; Altendorf et al., 2020). Improvement in traits including baking quality and disease resistance is also of interest (Bajgain et al., 2019b; Tyl & Ismail, 2019).

**Core Ideas**

- The two forms of shattering in intermediate wheatgrass are under independent genetic control.
- A QTL for brittle rachis type shattering was within 216 bp of an ortholog of *Btr2*.
- A floret shattering QTL on chromosome 11 is likely novel to intermediate wheatgrass.
- Similar to wheat, threshability QTL were found primarily on Group 2 chromosomes.
- Previous research on annual cereals has informed the domestication of intermediate wheatgrass.

Seed shattering is arguably the most significant of the domestication syndrome traits, as it marked the transition of a plant to dependence upon humans for seed dispersal and therefore reproductive success (Purugganan & Fuller, 2009). Nonshattering was one of the first traits to be selected in the ongoing domestication of American wild rice [*Zizania palustris* L. var. *interior* (Fassett) Dore] (Hayes et al., 1989; Kennard et al., 2002) and is suggested as an important criterion for selecting new candidate species for domestication (DeHaan et al., 2016). In perennial grasses, shattering can result in yield losses, narrowing the harvest timing window, and lead to stand contamination in perennial or nonrotational crops (Berdahl & Frank, 1998; Mueller‐Warrant & Rosato, 2002).

**FIGURE 1:**
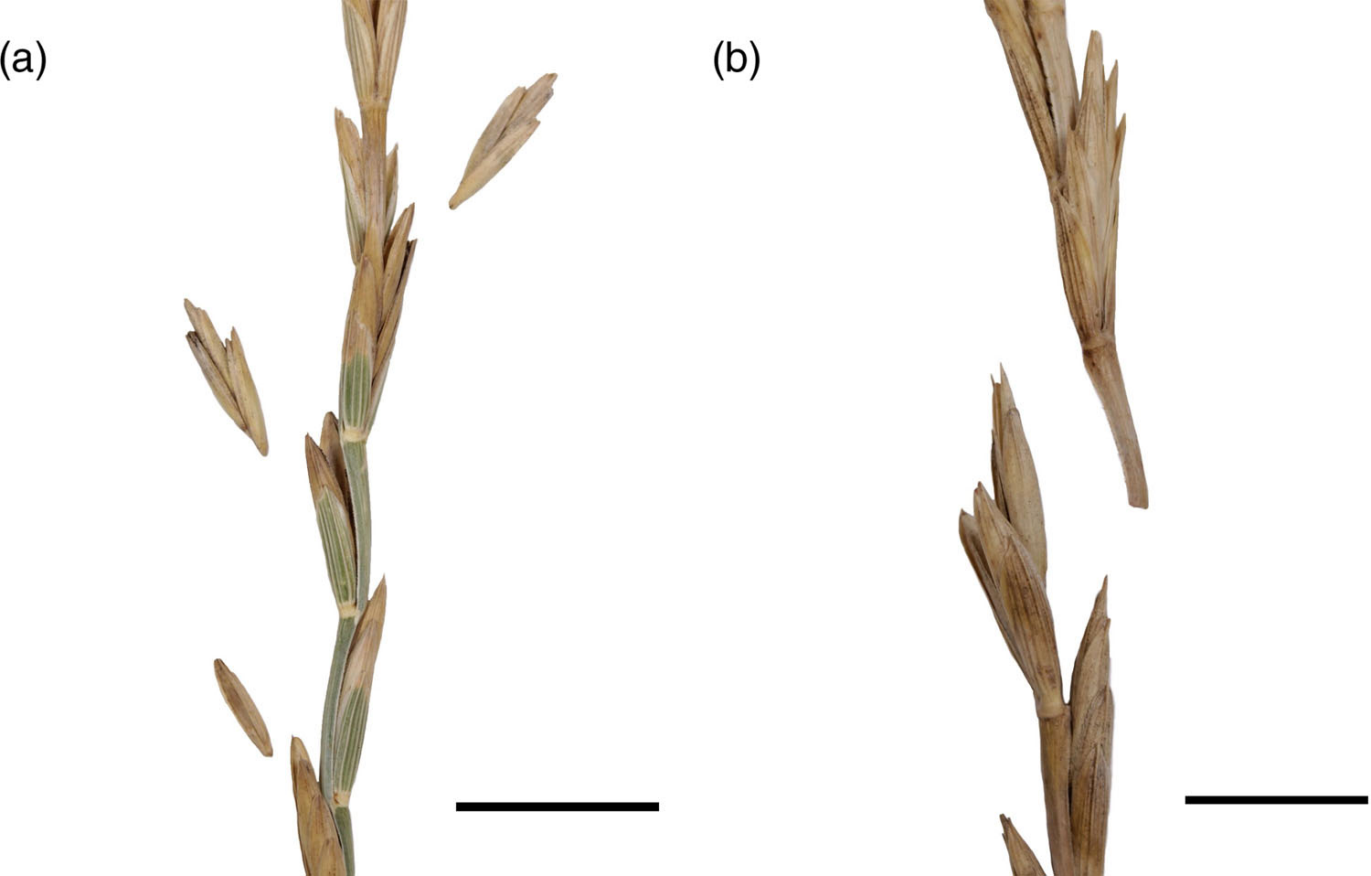
Images depicting the forms of seed shattering in intermediate wheatgrass: (a) floret shattering with disarticulation below rachilla node resulting in simple or complex caryopses with recessed rachilla internodes; (b) brittle rachis with disarticulation above rachis node producing wedge‐shaped caryopses including one or more spikelets with protruding rachis internode. Black bars are 1 cm

Intermediate wheatgrass has an elongated spike inflorescence and seed shattering can occur in two distinct ways (Larson et al., 2019). The first, which will be referred to as floret shattering, is characterized by disarticulation of florets along the rachilla axis below the rachilla node, which is the secondary axis of the spike. In this case, the dispersal unit is one or more caryopses that are each enclosed in a lemma and palea with a recessed rachilla internode above each point of disarticulation (Figure 1a). The second is wedge‐type brittle rachis, also referred to as spikelet shattering, where the disarticulation event occurs along the rachis above the spikelet attachment point. Here, a wedged‐shape dispersal unit includes one or more spikelets with a protruding rachis internode (Figure 1b). A third type, which is a second form of spikelet disarticulation, occurs when the rachis disarticulates below the spikelet node and results in a dispersal unit with recessed rachis internode (DeHaan et al., 2020). Floret shattering occurs in about half of the species in the Poaceae (Sakuma et al., 2011), whereas brittle rachis is unique to species in the Triticeae tribe (Pourkheirandish et al., 2015). It is speculated that floret shattering may be more ancient and more commonly associated with grasses with panicle inflorescence structure (Pourkheirandish et al., 2015). In barley, the brittle rachis trait is controlled by two genes, *Btr1* and *Btr2* on chromosome 3H, where a mutation at either locus confers a nonbrittle rachis (Pourkheirandish et al., 2015). In wheat, the recessive, nonbrittle rachis conferring alleles *Br‐A1* and *Br‐B1*, located on the short arm of chromosomes 3A and 3B, were fixed in domesticated emmer [*T. turgidum* L. subsp. *dicoccon* (Schrank) Thell.], the causal mutations for which were revealed in a sequence analysis in wild emmer [*T*. turgidum L. subsp. *dicoccoides* (Körn. ex Asch. & Graebn.) Thell.] (Watanabe et al., 2002; Avni et al., 2017). Nonbrittle rachis genes *btr1* and *btr2* also exist in einkorn wheat (*T. monococcum* L. subsp. *monococcum*) where a single nonsynonymous substitution at *btr1* results in the nonbrittle rachis trait (Pourkheirandish et al., 2018).

Selection for threshability was likely driven by an increase in harvesting efficiency. For example, in emmer wheat, the nonbrittle rachis and threshability traits together conferred an 85% reduction in time required for threshing (Tzarfati et al., 2013). The mechanism of threshability also varies depending on the species. For example, in non‐free‐threshing wheat, the caryopses are enclosed in tough, tenacious glumes that are difficult to separate from the spikelet to release the kernel (Kerber & Rowland, 1974). Whereas in barley, the lemma and palea adhere to the hull and it can also be difficult to separate the awns and rachis segments from the spikelet (Schmalenbach et al., 2011). In barley, this trait is controlled by the locus *nud* on chromosome 7HL (Taketa et al., 2008), in diploid wheat (*T. monococcum* L.) via soft glume (*sog* on 2A^m^S), and in polyploid wheat (*T. aestivum*) via tenacious glume (*Tg* on 2DS) (Sood et al., 2009) and the domestication gene *Q* on 5A (Simons et al., 2006). Although the lemma and palea of forage‐type IWG is tightly bound to the pericarp, rare genets, such as C3_3471, with high threshability characteristics, were identified after several cycles of selection (Larson et al., 2019).

We previously described a 1,168‐individual‐F_1_ nested association mapping (NAM) population of IWG that was developed from 11 phenotypically divergent parents from Cycle 2 of the University of Minnesota breeding program (Yu et al., 2008; Altendorf et al., 2021). In this previous work, we used flowering time traits as proof of concept and demonstrated that this population and the described methods were effective at identifying both new and previously detected quantitative trait loci (QTL) that align closely with well‐characterized flowering time orthologs from barley, notably *Ppd‐H1, Constans, and PhyB* (Altendorf et al., 2021). Here, we map three traits that are major targets of selection in IWG breeding: brittle rachis, floret shattering, and threshability. The objectives of this work were fourfold: (a) evaluate the phenotypic variation for these three domestication traits in the IWG NAM across four growing environments; (b) assess the relationship among these traits; (c) dissect their genetic control using two methods, genome‐wide association mapping (GWAS) and in linkage mapping both within and among families; and (d) compare QTL mapping results to potential domestication orthologs from related species.

## 2 MATERIALS AND METHODS

### 2.1 Population development and establishment

Parental selection and methods for developing the IWG NAM were previously described in detail in Altendorf et al. (2021). Briefly, 10 phenotypically diverse donor genets were identified from Cycle 2 of the UMN breeding program and crossed to WGN59, which is a low‐shattering genet, in a reciprocal manner. Approximately 130 seeds from each cross were germinated, allowed to tiller, and propagated into four ramets and transplanted at the University of Minnesota Agricultural Experiment Station in St. Paul, MN (STP hereafter), and in Salina, KS, at The Land Institute (TLI hereafter) in an randomized complete block design with two blocks each in fall 2016. The population was phenotyped during the 2017 and 2018 field seasons. At TLI in 2018, there was a severe drought, which resulted in plant death and poor performance of the plants and played a major role in the interpretation of the results in that environment.

### 2.2 Phenotypic data collection

Harvest timing and methods were also previously described in detail in Altendorf et al. (2020). Briefly, 10 of the larger, most uniform, and fully intact spikes from each spaced plant were cut ∼5 cm below the peduncle and placed in paper baguette bags. Uniform spikes were chosen not for selection but to reduce experimental error (DeHaan et al., 2018). Sample bags were carefully stored upright, peduncle‐down in cardboard boxes to limit shattering or breakage in the bag and were dried in a 35 °C dryer for 1 wk. Shattering was measured by selecting three unbroken, intact, and representative spikes and dropping them horizontally from 20‐cm height onto a 22.86‐ by 60.96‐cm (9‐ by 24‐inch) foam tray on an individual basis three times [method adapted from DeHaan et al. (2018); Supplemental Figure S1]. The number and type of disarticulation events were counted and recorded. Floret shattering, which occurs within the rachilla axis (Figure 1a), was quantified using the following scale: 1, 1–3 events; 2, 4–6 events; and increasing thereafter in increments of three. Brittle rachis disarticulation events, which occur within the rachis between spikelets, were simply counted (Figure 1b). The mean score for each type across the three spikes was calculated and rounded to the nearest integer. All shattered seeds, spike fragments, and spikes were returned to the bag and the 10‐spike sample was threshed on a Wintersteiger LD350 and cleaned as described in Altendorf et al. (2020). Threshability was estimated visually as the percentage of naked seeds by comparing with known standards in petri dishes. Samples were rated in increments of 5%, except for ratings between 90–100% and 0–10%, which were on 1% increments. To avoid the influence of moisture content of a spike on shattering and threshability, care was taken to ensure that samples were transferred from storage and placed back into a 35 °C dryer for at least 12 h prior to testing.

### 2.3 DNA variant detection and filtering

DNA variant detection methods were previously described in Altendorf et al. (2021). Genomic DNA was extracted from each genet using the BioSprint 96 Plant DNA Kit (QIAGEN) and 96‐plex genotyping‐by‐sequencing (Elshire et al., 2011; Poland et al., 2012) libraries were developed using a two‐enzyme (*Pst*I and *Msp*I), two‐barcode approach described in Zhang et al. (2016) and sequenced on Illumina HiSeq 2500. Reads were aligned to the v2 IWG reference genome (access provided by The *Thinopyrum intermedium* Genome Sequencing Consortium) using Bowtie2 v2.2.4 (Langmead & Salzberg, 2012) with the very‐sensitive presets. Reads that mapped uniquely were run through the Genome Analysis Toolkit v4.1.2 HaplotypeCaller, CombineGVCF, and GenotypeGVCF tools (McKenna et al., 2010). Markers were filtered in VCFTools v0.1.16 (Danecek et al., 2011) to include only biallelic SNPs with maximum 20% missing data, minimum allele depth of 5, and minor allele frequency >0.005 (0.05 per 10 families). Individuals were removed if they had >70% missing data or were determined to be selfs or unintended outcrosses as identified using Cervus v3.0 (Kalinowski et al., 2007). Marker imputation was conducted using LinkImpute v1.1.4 (Money et al., 2015).

### 2.4 Phenotypic data analysis

All data analyses were conducted in the program R v4.0.3 (R Core Team, 2020). Highly significant interactions between family and location, family and year, and location and year, in concert with differences in plant age, major climatic differences between environments, and a severe drought in one environment led to the analysis of each environment (location‐by‐year combination; *n* = 4) separately (Altendorf et al., 2020). Linear models were fit for each trait with fixed terms for genet nested within family and block (*n* = 2) as random. An arcsine square root transformation was applied to threshabilty to meet assumptions of ANOVA. For rachis breaks and floret score, where the count data was zero‐inflated, a generalized linear model was used. Estimated marginal means were calculated for genets nested within families on response and transformed scales as applicable using the ‘emmeans’ package (Lenth, 2018). Broad‐ and narrow‐sense heritabilities (*H* ^2^ and *h* ^2^, respectively) were calculated as described in Altendorf et al. (2021), across years separately for STP and TLI locations as these growing regions are targeted by separate breeding programs (Zhang et al., 2016; DeHaan et al., 2018). The drought conditions at TLI in 2018 confounded the variation in shattering and threshability, therefore heritability estimates were calculated from TLI 2017 data only. Pearson product moment correlations were conducted using the ‘corplot’ package (Wei et al., 2021). The primary objective of the correlation analysis was to assess relationships between brittle rachis, floret shattering, and threshability with yield component and maturity traits, and thus this analysis included some traits that are not featured in the present QTL analysis but were measured in the population. To assess whether phenotypically divergent parents yielded F_1_ families with high variability, the phenotypic difference between the parents was correlated with standard deviation among the progeny in each family. To evaluate the effects of the maternal parent, phenotpyic data from progeny derived from the common parent and donor parent as the mother were compared using a t‐test of unequal variance.

### 2.5 Genome‐wide association mapping and linkage mapping

Genome‐wide association mapping was conducted using the GAPIT software mixed linear model with the default kinship matrix calculation (Lipka et al., 2012). Markers with a *p* value below .00025 [logarithm of the odds (LOD) = 3.6] were considered significant. QTL within peaks were resolved using the ‘mmer’ function in the R package ‘Sommer’ (Covarrubias‐Pazaran, 2016) as described in Altendorf et al. (2021). Allele effects and frequencies were obtained from the GAPIT output. For GWAS QTL to be reported, they were required to be detected in at least two of the four environments (for reference, all GWAS QTL that were detected are featured in Supplemental Table S1). The NAM consensus genetic map was used for linkage mapping in MapQTL v 6 (Van Ooijen, 2009) using the two‐way pseudo testcross model using both the combined analysis and within‐family analysis approaches as described in Altendorf et al. (2021). Two maps were used, a map of the common parent and a map of the donor parents. A two‐LOD drop off interval was used to establish QTL intervals (Van Ooijen, 2009), which were identified using a custom R script. Allele effects and variance explained were calculated according to the MapQTL manual (Van Ooijen, 2009).

### 2.6 Visualization of results and proximity to orthologous genes

Marker position, both physical (Mbp) from the reference genome and genetic (cM) from the NAM consensus map across all chromosomes were normalized and visualized using ‘LinkageMapView’ (Ouellette et al., 2018). Markers in common between both the physical and the NAM genetic map were connected with a line. Intermediate wheatgrass chromosomes have shown high collinearity with barley, wheat (Zhang et al., 2016; Kantarski et al., 2017; DeHaan et al., 2020), and rice (homeologous chromosome groups are shown in Supplemental Table S2), and known domestication orthologs for shattering and threshability were previously aligned to the draft reference genome using a BLAST analysis (DeHaan et al., 2020; Altschul et al., 1997; Supplemental Table S3). These significant hits were used as reference positions for potential candidate genes in the QTL analyses.

### 2.7 Data availability

All scripts and raw data necessary to reproduce the analyses are available on the corresponding author's GitHub page (www.github.com/kraltendorf) in the repository IWG_NAM_Shattering_Threshability. Raw phenotype data, SNP genotype data, and the genetic map are available in digital repository for the University of Minnesota (DRUM; https://doi.org/10.13020/cnpv‐kf72). At the time of publication, the eleven parental clones from the NAM are being maintained at the University of Minnesota St. Paul Experimental Station in a long‐term parent nursery and clones are available upon request.

## 3 RESULTS

We evaluated two different mechanisms of seed shattering—brittle rachis and floret shattering—as well as seed threshability in the previously described IWG NAM population in four unique year‐by‐location environments (STP and TLI in 2017 and 2018). Both genome‐wide association mapping and linkage mapping within and across families were conducted for each of the three traits in the four unique environments. The authors refer the reader to our previous work (Altendorf et al., 2021) for additional detail on results from the creation and establishment of the population as well as variant detection analyses and genetic map creation.

### 3.1 Brittle rachis

**FIGURE 2:**
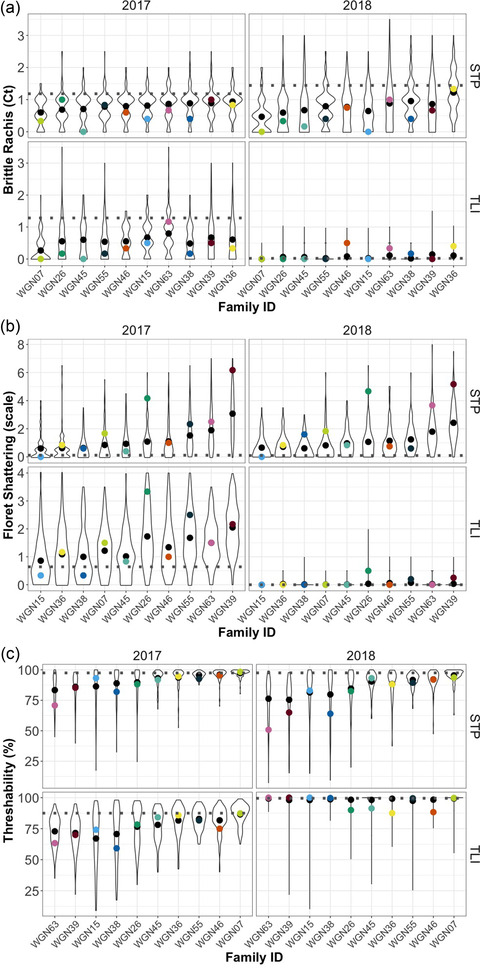
Performance of the 10 intermediate wheatgrass nested association mapping (NAM) families for: (a) brittle rachis (average count per three spikes per genet), (b) floret shattering (average rating across three spikes), and (c) threshability ratings for four distinct environments: St. Paul, MN (STP) and The Land Institute in Salina, KS (TLI) in 2017 and 2018. Within each trait plot, families are ordered by increasing progeny mean in STP 2017. Colored dots indicate parental means, black dots indicate progeny means. The horizontal gray dotted line indicates the mean of the common parent

**FIGURE 3:**
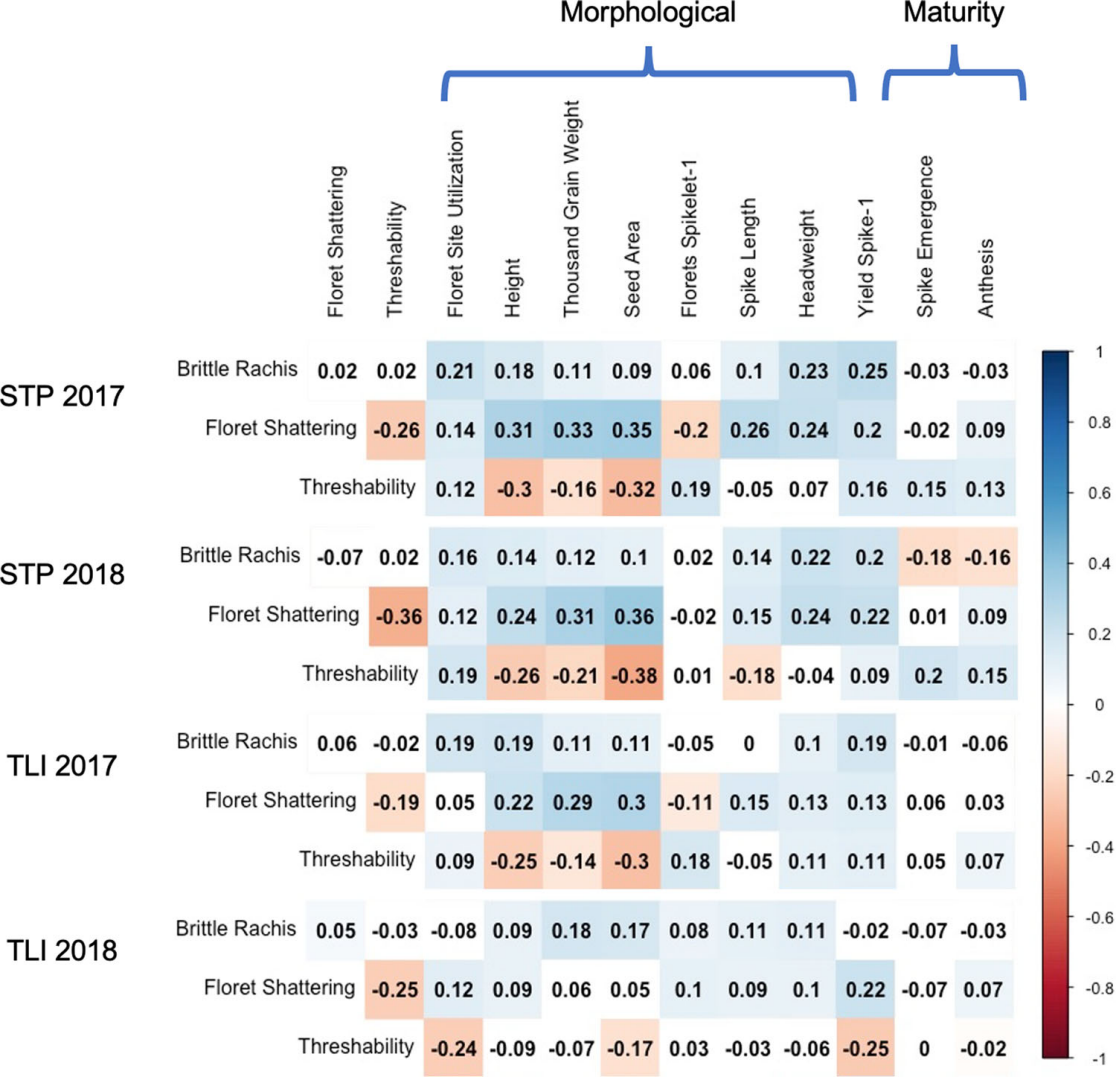
Pearson correlation coefficients between brittle rachis, floret shattering, and threshability with a series of morphological and maturity traits as observed in four unique environments where STP is St. Paul, MN, and TLI is The Land Institute in Salina, KS, in 2017 and 2018. All correlations shown were significant at the *r* = 0.01 threshold

**FIGURE 4:**
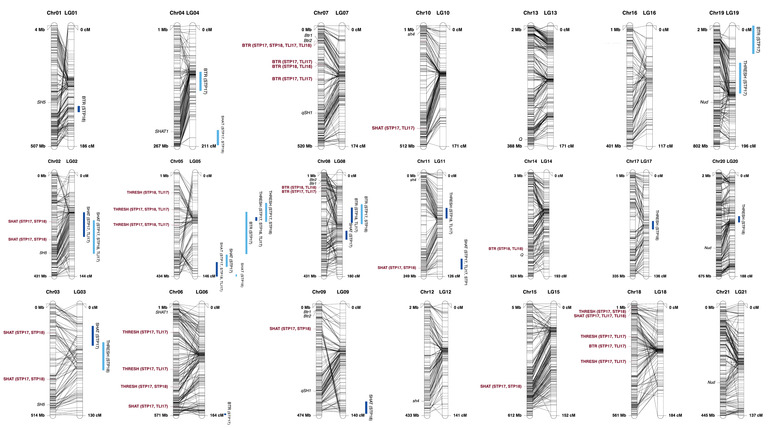
Results from the genome‐wide association study (GWAS) and linkage mapping (LM). For each chromosome, the physical map (unpublished, access provided by the *Thinopyrum intermedium* Genome Sequencing Consortium) is on the left, and linkage maps developed in the present study are on the right. Markers that were used in both mapping approaches are connected with a line. Physical distances in megabase pairs (Mbp) and genetic distances in centimorgans (cM) are normalized to comparable lengths. Included on the physical map (left), are the significant markers from genome‐wide association study (in maroon, bold), and possible candidate orthologous genes (black italic). Included on the linkage map (right) are the 2‐log of odds (LOD) drop off intervals for the combined analysis across populations (bars indicate interval length) for floret shattering (SHAT), brittle rachis (BTR), and threshability (THRESH), followed by the location (STP, St. Paul, MN; TLI, The Land Institute, Salina, KS), and years (17 and 18 for 2017 and 2018) in which the marker interval was detected. Intervals are colored by the map used for analysis, light blue for donor parent and dark blue for common parent. Further information on candidate orthologous genes can be found in Supplemental Table S3

Brittle rachis had the lowest heritability of the traits examined (*H* ^2^= 0.49, 0.42 and *h* ^2^ = 0.15, 0.13 at STP and TLI, respectively). Excluding TLI 2018, where brittle rachis occurred very rarely because of drought, the common parent, WGN59, was the most susceptible, exhibiting on average 1.2 rachis disarticulation events per spike tested (Figure 2a; Supplemental Table S4). Of the donor parents, WGN63, WGN36, and WGN39 were the most susceptible, while WGN07 was the most resistant and the progeny means of these families followed this trend and displayed evidence for transgressive segregation (Figure 2a). Phenotypic difference between the parents, however, was only a significant predictor of progeny variation in one of four environments (Supplemental Table S5). Brittle rachis did not share any notable (absolute value *r* < 0.3) correlations with the traits surveyed (Figure 3). In the GWAS analysis, four QTL were detected for brittle rachis on chromosomes 7, 8, 14, and 18. While they explained only a small percentage (avg 1.5%) of the total variation, they were detected across at least two of the four environments (specific environment was variable depending on the QTL) with the exception of Chr07_66140981, which was detected in all four environments (Figure 4; Table 1; Manhattan plots in Supplemental Figure S2). One QTL was in common between the two analyses, on chromosome 8, which was segregating in both the common and donor parents (Figure 4; Table 2; within family effects are in Supplemental Table S6) and explained on average 17% (range 12–23%) of the variation, depending on the family and environment. The major GWAS QTL region on chromosome 7 was not detected in linkage mapping, but others in the combined analysis were found on chromosomes 1, 4, 5, 6, and 19 (Table 2). Results from all ANOVA are included in Supplemental Table S7. Within‐location means for each trait are in Supplemental Table S8.

**TABLE 1:** Results from genome‐wide association mapping results for all environments organized by trait and environment (top row). Instances in which the quantitative trait loci (QTL) was not detected in the present environment are indicated by missing values (–)

					STP 2017			STP 2018			TLI 2017			TLI 2018		
Trait	Chr	Position	Alleles^a^	MAF^b^	−log10(p)^c^	Effect^d^	PVE^e^	−log10(p)	Effect	PVE	−log10(p)	Effect	PVE	−log10(p)	Effect	PVE
		bp														
Brittle rachis	7	66,140,981	A/G	0.07	8.93	0.3	0.03	6.88	0.37	0.03	10.63	0.41	0.05	6.04	0.1	0.0181
	7	166,602,183	G/A	0.05	5.09	0.29	0.00	–	–	–	5.65	0.39	0.00	–	–	–
	7	167,995,817	A/C	0.05	–	–	–	5.07	‐0.43	0.00	–	–	–	4.67	‐0.11	0.0136
	7	230,540,646	T/C	0.05	4.1	0.24	0.00	–	–	–	4.8	0.32	0.00	–	–	–
	7	353,992,030	T/C	0.05	5.99	‐0.3	0.00	–	–	–	5.65	‐0.36	0.00	–	–	–
	8	57,185,788	T/A	0.07	–	–	–	3.7	0.26	0.00	–	–	–	4.31	0.09	0.0025
	8	77,402,910	G/A	0.46	4	‐0.11	0.04	–	–	–	3.84	‐0.14	0.02	–	–	–
	14	386,756,967	G/A	0.07	–	–	–	4.34	0.3	0.02	–	–	–	4.55	0.09	0.0051
	18	214,242,119	G/A	0.05	5.13	0.29	0.02	–	–	–	7.2	0.44	0.07	–	–	–
Floret shattering	2	204,450,791	C/T	0.03	5.24	1.26	0.49	4.01	0.9	0.05	–	–	–	–	–	–
	2	278,677,992	C/A	0.45	8.91	‐0.47	0.03	3.63	‐0.24	0.02	–	–	–	–	–	–
	3	133,897,959	G/A	0.03	4.89	1.18	0.01	3.96	0.87	0.00	–	–	–	–	–	–
	3	347,078,225	C/T	0.03	5.03	1.19	0.00	3.8	0.84	0.00	–	–	–	–	–	–
	6	524,746,973	T/C	0.37	8.38	‐0.56	0.02	–	–	–	5.3	‐0.32	0.01	–	–	–
	9	106,857,528	G/A	0.03	4.35	1.11	0.03	4.61	0.95	0.08	–	–	–	–	–	–
	10	436,139,244	A/G	0.46	4.79	0.32	0.00	–	–	–	4.74	0.23	0.01	–	–	–
	11	228,804,489	T/G	0.42	5.71	‐0.29	0.06	4.23	‐0.2	0.03	–	–	–	–	–	–
	15	454,588,273	C/A	0.05	6.83	2.18	0.22	5.85	1.66	0.07	–	–	–	–	–	–
	18	40,836,512	G/A	0.46	5.11	‐0.57	0.01	–	–	–	3.8	‐0.36	0.00	5.12	‐0.07	0.0088
Threshability	5	86,058,515	G/A	0.35	–	–	–	6.26	‐0.07	0.01	11.39	‐0.09	0.03	–	–	–
	5	156,358,046	G/T	0.35	12.36	‐0.09	0.06	11.03	‐0.1	0.05	12.72	‐0.1	0.06	–	–	–
	5	219,902,957	C/T	0.45	8.21	‐0.07	0.02	–	–	–	–	–	–	–	–	–
	5	219,902,957	C/T	0.46	–	–	–	8.39	‐0.08	0.02	7.52	‐0.07	0.01	–	–	–
	6	147,094,010	C/G	0.27	5.11	0.07	0.02	–	–	–	4.5	0.07	0.02	–	–	–
	6	334,530,477	C/T	0.27	5.23	0.08	0.01	–	–	–	3.63	0.07	0.01	–	–	–
	6	424,161,336	T/A	0.05	6.19	‐0.11	0.01	4.85	‐0.11	0.01	–	–	–	–	–	–
	18	17,030,514	G/A	0.09	3.98	‐0.06	0.03	5.82	‐0.09	0.03	–	–	–	–	–	–
	18	165,923,396	C/A	0.08	4.91	‐0.08	0.03	–	–	–	4.06	‐0.08	0.01	–	–	–
	18	291,366,356	G/A	0.08	4.14	‐0.07	0.00	–	–	–	3.68	‐0.07	0.00	–	–	–

^a^ Reference and alternate alleles.

^b^ MAF, minor allele frequency within the entire 10‐family nested association mapping population.

^c^ Level of significance.

^d^ Allele effect in trait units, associated with the alternate allele.

^e^ PVE, percentage variance explained by the QTL.

**TABLE 2:** Results from linkage mapping analyses combined across families for brittle rachis, floret shattering, and threshability including the peak centimorgan (cM) position, locus, and max log of odds (LOD) across three environments. Note, no significant quantitative trait loci (QTL) were detected in the combined analysis at The Land Institute (TLI) 2018 for these traits. Results for within‐family analyses including variance explained and allele effects are in Supplemental Table S1

			STP 2017			STP 2018			TLI 2017		
Trait	Chr^a^	Map^b^	Peak position^c^	Peak locus^d^	Max LOD^e^	Peak position	Peak locus	Max LOD	Peak position	Peak locus	Max LOD
			cM			cM			cM		
Brittle rachis	1	CP	–	–	–	126.6	CP_Chr01_410726049	12.71	–	–	–
	4	DP	83.9	DP_Chr04_220417527	9.42	–	–	–	–	–	–
	5	DP	83.1	–	17.3	–	–	–	–	–	–
	6	CP	163.1	CP_Chr06_529546708	15.32	–	–	–	–	–	–
	8	CP	–	–	–	68.7	CP_Chr08_23347234	9.73	75.6	CP_Chr08_22429423	11.85
	8	DP	82.0	–	8.14	82.0	–	11.08	–	–	–
	19	DP	32.6	–	17.94	–	–	–	–	–	–
Floret shattering	2	CP	76.8	CP_Chr02_343055305	7.98	–	–	–	77.5	CP_Chr02_338749407	12.37
	2	DP	85.0	–	9.16	91.4	–	8.65	77.9	–	9.21
	3	CP	30.9	CP_Chr03_78858449	7.95	–	–	–	–	–	–
	4	DP	196.5	–	14.68	195.5	DP_Chr04_260663237	12.21	–	–	–
	5	CP	135.0	–	18.4	136.0	–	16.03	136.0	–	16.89
	5	DP	127.8	–	8.09	146.4	DP_Chr05_339720810	9.36	–	–	–
	8	CP	108.7	CP_Chr08_303295574	7.8	–	–	–	–	–	–
	9	CP	–	–	–	131.9	CP_Chr09_111456911	11.25	–	–	–
	11	CP	109.2	–	31.98	109.2	–	35.04	109.2	–	21.73
Threshability	3	DP	–	–	–	64.5	DP_Chr03_287042026	11.18	–	–	–
	5	CP	65.8	CP_Chr05_184445451	45.77	65.5	CP_Chr05_111147787	47.04	65.8	CP_Chr05_184445451	60.18
	5	DP	62.8	DP_Chr05_196581222	10.81	62.8	DP_Chr05_196581222	10.51	–	–	–
	11	CP	–	–	–	46.8	CP_Chr11_34860853	10.77	51.3	–	12.06
	17	CP	–	–	–	69.4	CP_Chr17_228138417	8.45	–	–	–
	19	DP	88.9	DP_Chr19_131389206	8.84	–	–	–	–	–	–
	20	CP	–	–	–	85.1	CP_Chr20_437898043	8.09	–	–	–

^a^ Chr, chromosome.

^b^ Map used in linkage mapping; CP, common parent; DP, donor parent.

^c^ Genetic position in centimorgans (cM) of the 2‐LOD drop off peak interval.

^d^ Peak locus of the QTL interval where dash (–_ indicates that the peak was between two markers.

^e^ Max LOD for that locus or position.

### 3.2 Floret shattering

Floret shattering was moderately heritable (*H* ^2^= 0.83, 0.65 and *h* ^2^ = 0.52, 0.34 at STP and TLI, respectively). WGN59 ranked among the most resistant, while WGN39 and WGN26 exhibited a respective average of 4.5 and 4.06 on the scale, which is equivalent to 13.5 and 12.2 floret disarticulation events per spike tested (Figure 2b; Supplemental Table S4). The phenotypic difference between parental phenotypes was a significant predictor of progeny variation in all four environments (*P* < .1; Supplemental Table S5). It shared a moderate, significant correlation with seed area and 1000‐grain weight (*r* across environments, excluding TLI 2018, ranged from 0.29 and 0.36; Figure 3). Floret shattering had the highest number of QTL of all traits reported here, including 10 QTL across eight chromosomes in GWAS and seven significant regions in linkage mapping, suggesting highly polygenic control (Tables 1 and 2; Manhattan plots in Supplemental Figure S3). Chromosomes 2, 3, 9, and 11 were in common across the two analyses. The QTL on chromosome 11, which explained 5.8 and 3.5% of the variation in the GWAS analysis where it was detected, segregated in the common parent and the alternate allele had a consistent, negative effect, corresponding to the distribution of progeny and parent phenotypic means for this trait (Figure 2b). Linkage mapping identified two other significant regions on chromosomes 4 and 5, which were not detected in GWAS (Figure 4; Tables 1 and 2; Supplemental Table S6).

### 3.3 Threshability

Threshability, or percentage dehulled seed, was highly heritable (*H* ^2^= 0.91 and 0.91 and *h* ^2^ = 0.49 and 0.40 at STP and TLI, respectively) and was generally high across the entire NAM population (Figure 2c). Excluding TLI 2018 where variation in threshability was confounded by low seed number as a result of drought conditions, WGN59 had the highest threshability (average across environments = 96%) and WGN63 had the lowest threshability (71%), and progeny values followed a similar trend. Variation in the parents was a significant predictor of progeny standard deviation in three of four environments (*P* < .1; Supplemental Table S1 and there was no effect of maternal parent (avg. *P* = .44; Supplemental Table S9). Threshability and seed area were negatively correlated (range in *r* = −0.17 to −0.38; Figure 3). Threshability QTL were detected on chromosomes 5, 6, and 18 in GWAS and chromosomes 3, 5, 11, 17, 19, and 20 in linkage mapping (Figure 4; Tables 1 and 2; Manhattan plots in Supplemental Figure S4). The GWAS marker S05_156358046 was the most significant of all QTL detected in this study (−log_10_(*p*) ranging from 11 to 12.7) and explained a relatively high percentage of the variation (range in percentage variance explained = 5.3–6.4%) and was consistently detected in STP 2017, STP 2018, and TLI 2017. Two other markers were detected in chromosome 5, and while they were physically distant, they align to a similar region in the consensus genetic map and overlap with a QTL interval in linkage mapping. This region was detected in the analysis of both the common parent (three of four environments) and donor parent (two of four environments) WGN15 and explained 10–21% of the variation (Table 2; within‐family effects in Supplemental Table S6).

## 4 DISCUSSION

In several crop species, domestication syndrome traits are under the control of single or few large‐effect QTL (e.g. Simons et al., 2006; Taketa et al., 2008; Asano et al., 2011). Considering the highly conserved gene order across species in the Triticeae tribe and the decades of research into understanding the genetics of domestication, it may be possible to use this knowledge to expedite the domestication of new crops such as in the case of IWG (DeHaan et al., 2020). Using a large NAM population of IWG to investigate the genetic control of seed shattering and threshability traits, we demonstrated that QTL for brittle rachis, floret shattering, and threshability are either physically close or on the same chromosome or homeologous groups as significant BLAST hits to major domestication orthologous genes in barley, wheat, and rice. For example, IWG chromosomes 7 and 8 are a part of Group 3, which is collinear with barley chromosome 3H, where *Btr1* and *Btr2* are located (DeHaan et al., 2020). In particular, the chromosome 7 marker (Chr07_66140981), which was detected in all four environments, was within 216 bp from a significant BLAST hit of *Btr2*, and within <1.44 Mbp of five *Btr1‐* or *Btr1*‐like genes (Table 1; Supplemental Table S3). On chromosome 8, markers Chr08_57185788 (detected in two of four environments) and Chr08_ 77402910 (two of four environments) were within 19 and 39 Mbp of BLAST hits of *Btr1* and *Btr2*. In linkage mapping on chromosome 8, the 2‐LOD drop off interval for a QTL found at two of four environments peaked between 68.7 and 82 cM and overlapped with this same region (Figure 4; Table 1; Candidate orthologous gene positions in Supplemental Table S3). The GWAS hit for brittle rachis on chromosome 14, which was detected in two environments, was 31 Mbp away from a BLAST hit for the domestication gene, *Q*, of wheat, which is known to affect rachis fragility (DeHaan et al., 2020; Simons et al., 2006). This region, however, was not detected using linkage mapping.

For floret shattering, the peak locus for the QTL segregating in the common parent on chromosome 2 (CP_Chr02_343055305 in STP 2017 and CP_Chr02_338749407 in TLI 2017; Table 2), aligned within 4.3 and 8.6 Mbp of a candidate ortholog BLAST hit for *SH5*, which enhances seed shattering in rice (DeHaan et al., 2020; Yoon et al., 2014). The floret shattering QTL on chromosome 11 aligns closely with a previously reported QTL in IWG for shattering (Larson et al., 2019). Interestingly, a significant BLAST hit for the candidate ortholog, *sh4* of rice, was on the opposite end of chromosome 11 (DeHaan et al., 2020; Li et al., 2006). Thus, the shattering QTL on chromosome 11 seems to be a novel gene that is potentially unique to IWG. Moreover, another shattering QTL is located on a similar region of homeologous chromosome 10, providing potential evidence for two functional copies of this unknown causative gene across homeologous chromosomes. On chromosome 4, a BLAST hit for the candidate ortholog of rice, *SHAT1*, is within 26 Mbp of the peak LOD marker for floret shattering, which was detected in two environments (DeHaan et al., 2020; Zhou et al., 2012).

While previous researchers have defined the mechanisms of seed shattering in IWG (Larson et al., 2019; DeHaan et al., 2020), this study is the first to distinguish between brittle rachis and floret shattering in phenotyping and data analysis. To do this, we adapted a spike drop test method described by DeHaan et al. (2018) and expanded the scale to characterize and record the two main types of disarticulation events separately. The phenotypic correlation analysis demonstrated that there was only a very weak correlation (ranging from −0.07 to 0.06, depending on environment) between the two types of shattering, providing evidence for independent genetic control (Figure 3). Furthermore, neither trait showed a strong correlation with maturity traits. While floret shattering was moderately correlated with seed size, correlations with other traits that might exhibit a mechanical influence on shattering were minimal. This finding, in combination with high heritability estimates, suggests that the variation primarily was due to genetics and less‐so to environmental variation, early maturity, or mechanical influences. The QTL mapping results showed that the two are indeed likely under distinct genetic control in this population of IWG, with limited evidence for shared QTL between the traits. Quantitative trait loci for both traits exist on chromosomes 5 and 8, though they appear to be in distinct regions. The QTL detected for floret shattering correspond to others detected in Larson et al. (2019) on chromosomes 6, 10, 11, and 12. The Larson et al. (2019) study, however, did not detect seed shattering QTL on homoeologous Group 3 (chromosomes 7 and 8), where the *Btr* orthologs are located. It is possible this trait was not segregating in their M26 × M35 IWG biparental population, or the method of phenotyping did not reveal the variation for this trait. According to the results described here, this variant was still segregating in C2 of the UMN program from which this population was derived. The relatively simple genetic control would suggest that it may be easy to purge with the correct method of phenotyping and selection. Floret shattering, with its more complex genetic control, may be more difficult to purge, but this may be overcome with more precise phenotyping and selection, as well as incorporating significant markers identified here in routine genomic selection pipelines as fixed effects (Bajgain et al., 2019a).

The mechanism for threshability varies depending on the species, and in IWG, threshability has historically been compared with barley, where seeds are hulled, and not like wheat where the caryopses are difficult to remove from the glumes. Previous work has suggested that the barley crop evolution gene for seed nakedness, *nud*, is a putative candidate ortholog for the threshability in IWG (Larson et al., 2019; DeHaan et al., 2020). Interestingly, no threshability QTL were detected on homeologous Group 7 chromosomes, where the BLAST hits were (Supplemental Table S3). Only very rarely did we observe seeds with lemma and palea physically adhered to the caryopsis, as in this population they could be removed by hand with relative ease. Since the UMN breeding program was derived from a small subset of improved material from the TLI program (Zhang et al., 2016) and this population represents an even smaller portion of that variation, it is possible that the adherent hull characteristic was not segregating in the NAM. We did, however, detect large‐effect QTL on Group 2 chromosomes (Table 1), specifically chromosome 5, notably where the wheat soft glume (*sog)* and tenacious glume (*Tg)* loci are located (Sood et al., 2009). In contrast to the adherent hull trait of *nud*, this mechanism is characterized by easier removal of the caryopsis from the glumes. In this study, threshability was rated as percentage clean seed after threshing in a Wintersteiger LD350, in which spikes are broken and hulls removed by rubber beaters rapidly oscillating against a metal concave in a drum. Overall, threshability was very high across the population and whether this was due to the population itself having high threshability or was due to the method used is not clear. It is possible that if the caryopses are easier to remove from the glumes, they spend more time in the threshing drum before they are released through the concave, giving more opportunity for lemma and palea removal, and vice versa for caryopses that require more force to release from the glumes. Furthermore, as evidenced by the tendency for IWG to exhibit floret shattering, its rachilla axis is fragile and long, sometimes containing numerous florets (up to 12; Altendorf et al., 2020). There was a negative correlation between threshability and floret shattering (*r* = −0.19 to −0.36), suggesting that individuals with greater shattering resistance had greater percentage naked seed, a finding also reported by Larson et al. (2019). An alternative hypothesis is that spikes with a more rigid rachilla axis with lower floret shattering retain the lemma and palea intact to the spike within the thresher, releasing a higher proportion of naked seeds. In the case of rice, fine tuning of the expression of the shattering genes *Sh3* and *Sh4* was important for maintaining low shattering but also high threshability of the grain (Li et al., 2006; Lv et al., 2018), and a similar approach may be required for shattering or threshability genes in IWG. Further sequence similarity analysis across wheat and IWG using the simple sequence repeat markers linked with *Tg* and *sog* (these genes have yet to be cloned; Haas et al., 2019) in wheat would need to be conducted to provide further evidence for this hypothesis in addition to further study into the mechanics of threshability in IWG.

The GWAS QTL detected in multiple environments are not supported by linkage mapping results in every case. In general, the GWAS analysis paints a much more complex picture of the genetic control of the shattering and threshability traits than does the linkage mapping analysis. This could be due to a few reasons. First, linkage mapping may have been limited in its effectiveness because of the severe segregation distortion observed in the linkage map creation process (Altendorf et al., 2021). Because of the severe segregation distortion and singularity errors associated with long spans of uninformative markers, we were required to use the two‐way pseudo testcross method, which excludes any markers that are heterozygous in both parents and uses only markers that are heterozygous in one and homozygous in the other, further reducing the marker count. It is possible that some QTL were missed as a result of low marker density. For example, it is puzzling that GWAS for brittle rachis detected QTL on chromosome 7 in all four environments, even in TLI 2018 where brittle rachis barely occurred at all under the severe drought conditions, but this region was not detected in the linkage mapping analysis. We previously reported marker types and counts for the linkage map creation step, and chromosome 7 had an especially low number of informative markers (Altendorf et al., 2021). While the Chr07_66140981 marker was the most significant in all four environments, Manhattan plots of the GWAS results for brittle rachis in all four environments do not reveal a distinct peak, instead, there are significant markers across the length of the chromosome (Supplemental Figure S2). While the rate of linkage disequilibrium decay on chromosome 7 was close to the median across all chromosomes (*r* ^2^ = 0.2 at 21.1 cM; Altendorf et al., 2021), it is possible that rate of effective recombination in this population was low. Second, the v2 IWG genome is considered a draft and there are various errors, several of which are visible in the discrepancy of the ordering of markers on the genetic and physical maps (Figure 4). It is also possible that there are errors in the placement of markers on different chromosomes within homoeologous groups and perhaps even across homoeologous groups resulting in what may appear to be spurious significant associations in GWAS, which is the reason we chose to only report GWAS markers that were significant in more than one environment.

In the development of biparental populations for QTL mapping, it is common to select individuals that vary phenotypically for a trait of interest. Here we showed that divergent parents only consistently yielded divergent progeny in the cases of floret shattering and threshability, whereas brittle rachis varied depending on the environment. In an outcrossing, heterozygous species like IWG it is of equal importance to choose highly heterozygous parents in order to observe segregation in the progeny. In several cases, significant and large‐effect QTL were detected in families where the parents exhibited very similar phenotypic values. These parents were likely heterozygous for several important QTL. One example is threshability in family WGN15, where the parents performed similarly but progeny were highly variable (Figure 2c), and QTL were consistently detected on chromosome 5 and explained a large proportion of the variance in that family.

Previous work in IWG has demonstrated the potential for using significant QTL markers as fixed effects in genomic selection to increase the frequency of beneficial alleles (Zhang et al., 2017; Bajgain et al., 2019a). Prediction accuracy in genomic selection in IWG has been reportedly higher for domestication traits such as shattering and threshability compared with agronomic traits (Crain et al., 2020). In floret shattering, where the trait appears to be under more complex genetic control, genomic selection may be a viable solution for improvement. In cases of traits like brittle rachis and threshability, where the genetic control appears to be less complex, working toward the validation of these QTL and developing resources for marker‐assisted selection may allow breeders to select parental material with advantageous alleles for crossing blocks. An alternative and potentially complementary approach may be exploring the use of targeted mutagenesis in known domestication gene sequences to alter gene function (DeHaan et al., 2020), and efforts to do so may be better informed by the results of this study that provides substantial evidence in support of potential candidate orthologous genes. Finally, a robust phenotyping method that separates the two types of shattering and allows the breeder to select for resistance independently may be more precise and lead to rapid gains. This study demonstrates a case where decades of research into the domestication history of our major cereal crops has direct applications in the domestication of new, perennial grain crops that may serve as one way for modern plant scientists to improve the sustainability of agriculture.

## CONFLICT OF INTEREST

The authors report no conflict of interest.

## AUTHOR CONTRIBUTIONS

Kayla R. Altendorf: Conceptualization; Data curation; Formal analysis; Investigation; Methodology; Project administration; Visualization; Writing‐original draft; Writing‐review & editing. Lee R. DeHaan: Conceptualization; Project administration; Supervision; Writing‐review & editing. Steve R. Larson: Formal analysis; Methodology; Writing‐review & editing. James A. Anderson: Funding acquisition; Project administration; Resources; Writing‐review & editing.

## Supporting information


**Supplemental Table S1**. All quantitative trait loci (QTL) detected using genome‐wide association study (GWAS) for each of the three traits in only one environment where STP = St. Paul, MN and TLI = The Land Institute in Salina, KS in 2017 and 2018. Only QTL detected in two or more of the four environments are reported in the main text.
**Supplemental Table S2**. Homeologous chromosomes in intermediate wheatgrass and their colinear relationship to chromosomes in barley.
**Supplemental Table S3**. Candidate domestication orthologs, their marker name as shown in Figure 4 with the chromosome (Chr) and position (Pos) of significant BLAST hits in intermediate wheatgrass version 2 draft genome (access provided by the Intermediate Wheatgrass Genome Sequencing Consortium) as reported by DeHaan et al., 2020
**Supplemental Table S4**. Mean phenotypic values for the parents of the intermediate wheatgrass Nested Association Mapping (NAM) population across traits all traits and environments, where STP = St. Paul, MN and TLI = The Land Institute in Salina, KS in 2017 and 2018.
**Supplemental Table S5**. Pearson correlation estimates and p‐values from analysis of the difference between parental phenotypic values and the standard deviation among their progeny.
**Supplemental Table S6**. Results from linkage mapping analyses combined across families and within families for brittle rachis, floret shattering, and threshability, including two‐LOD drop off intervals, peak loci, maximum LOD, variance explained and allele effects.
**Supplemental Table S7**. Results from analysis of variance for mixed effects linear models within each environment for each trait
**Supplemental Table S8**. Minimum, mean, maximum and standard deviation for phenotypic values within environments where STP = St. Paul, MN and TLI = The Land Institute in Salina, KS in 2017 and 2018 for each trait.
**Supplemental Table S9**. Mean of progeny derived from the common parent (C) or the donor parent (D) as the mother within families of the intermediate wheatgrass nested association mapping population across environments for each trait. P‐value is the result from a t‐test with unequal variance between the two progeny types.
**Supplemental Figure S1**. Shattering drop test method. Three intermediate wheatgrass spikes were dropped horizontally from 20 cm height three times onto a foam tray and disarticulation events were characterized, counted and recorded.
**Supplemental Figure S2**. Manhattan plots for genome‐wide association mapping of brittle rachis type shattering across four environments (STP = St. Paul, MN; TLI = The Land Institute in Salina, KS). Horizontal gray dotted line indicates significance threshold of LOD = 3.6.
**Supplemental Figure S3**. Manhattan plots for genome‐wide association mapping of floret shattering across four environments (STP = St. Paul, MN; TLI = The Land Institute in Salina, KS). Horizontal gray dotted line indicates significance threshold of LOD = 3.6.
**Supplemental Figure S4**. Manhattan plots for genome‐wide association mapping of threshability across four environments (STP = St. Paul, MN; TLI = The Land Institute in Salina, KS). Horizontal gray dotted line indicates significance threshold of LOD = 3.6.
