# Supplementary material for: QTL for seed shattering and threshability in intermediate wheatgrass align closely with well‐studied orthologs from wheat, barley, and rice
Source: Plant Genome. 2021 Oct 9;14(3):e20145. doi: 10.1002/tpg2.20145 (PMC12806861; doi:10.1002/tpg2.20145)
Supplement: Supplementary file 1 [file TPG2-14-e20145-s001.docx]

**QTL for seed shattering and threshability in intermediate wheatgrass align closely with well-studied orthologs from wheat, barley, and rice**

Kayla R. Altendorf, Lee R. DeHaan, Steve R. Larson, James A. Anderson

USDA-ARS Forage Seed and Cereal Research Unit, Prosser, WA 99350; The Land Institute, Salina, KS 67401; USDA-ARS Forage & Range Research Lab, Logan, UT 84322; Department of Agronomy and Plant Genetics, University of Minnesota, St. Paul, MN 55108

**Supplementary Tables and Figures:**

**Table S1.** All quantitative trait loci (QTL) detected using genomewide association study (GWAS) for each of the three traits in only one environment where STP = St. Paul, MN and TLI = The Land Institute in Salina, KS in 2017 and 2018. Only QTL detected in two or more of the four environments are reported in the main text.

|  |  |  |  |  | STP 2017 | | | STP 2018 | | | TLI 2017 | | | TLI 2018 | | |
| --- | --- | --- | --- | --- | --- | --- | --- | --- | --- | --- | --- | --- | --- | --- | --- | --- |
| Trait | Chr | Pos | Alleles | MAF | -log10(p) | Allele Effect | PVE | -log10(p) | Allele Effect | PVE | -log10(p) | Allele Effect | PVE | -log10(p) | Allele Effect | PVE |
| Brittle Rachis | 1 | 77245973 | A/T | 0.04 | - | - | - | - | - | - | - | - | - | 4.29 | 0.11 | 0.01 |
|  | 1 | 405808201 | G/C | 0.42 | - | - | - | 5.26 | 0.2 | 0.05 | - | - | - | - | - | - |
|  | 2 | 143430796 | G/A | 0.05 | - | - | - | - | - | - | - | - | - | 4.16 | 0.1 | 0.01 |
|  | 4 | 186994203 | C/G | 0.16 | 4.47 | -0.18 | 0.01 | - | - | - | - | - | - | - | - | - |
|  | 4 | 262582812 | A/T | 0.05 | - | - | - | - | - | - | - | - | - | 4.18 | 0.11 | 0.01 |
|  | 6 | 394909182 | C/T | 0.21 | 4.18 | -0.16 | 0.01 | - | - | - | - | - | - | - | - | - |
|  | 6 | 396830702 | T/C | 0.04 | - | - | - | - | - | - | - | - | - | 3.86 | 0.1 | 0.02 |
|  | 6 | 532845545 | G/A | 0.04 | - | - | - | 4.36 | 0.34 | 0.02 | - | - | - | - | - | - |
|  | 7 | 63220880 | G/A | 0.04 | - | - | - | 4 | 0.3 | 0.01 | - | - | - | - | - | - |
|  | 7 | 66140981 | A/G | 0.07 | 8.93 | 0.3 | 0.04 | - | - | - | - | - | - | - | - | - |
|  | 7 | 120924755 | C/T | 0.03 | - | - | - | - | - | - | 3.97 | 0.41 | 0.02 | - | - | - |
|  | 7 | 172148769 | T/A | 0.49 | - | - | - | - | - | - | - | - | - | 4.39 | 0.05 | 0.01 |
|  | 7 | 271265676 | A/C | 0.05 | - | - | - | 5.07 | -0.43 | 0.06 | - | - | - | - | - | - |
|  | 7 | 350460622 | G/A | 0.08 | 3.67 | -0.18 | 0 | - | - | - | - | - | - | - | - | - |
|  | 7 | 350585028 | G/C | 0.4 | - | - | - | - | - | - | 4.56 | 0.18 | 0 | - | - | - |
|  | 8 | 36544509 | C/T | 0.15 | - | - | - | - | - | - | - | - | - | 3.74 | 0.07 | 0.01 |
|  | 9 | 147855386 | C/G | 0.36 | - | - | - | - | - | - | 3.63 | -0.17 | 0.01 | - | - | - |
|  | 10 | 436139188 | G/A | 0.06 | - | - | - | - | - | - | - | - | - | 5.42 | 0.1 | 0.01 |
|  | 16 | 49416735 | G/A | 0.02 | - | - | - | - | - | - | - | - | - | 4.25 | 0.14 | 0.02 |
|  | 18 | 214242119 | G/A | 0.05 | - | - | - | - | - | - | 7.2 | 0.44 | 0.04 | - | - | - |
|  | 19 | 641594491 | C/T | 0.02 | - | - | - | - | - | - | 3.72 | -0.38 | 0 | - | - | - |
|  | 20 | 5976092 | C/T | 0.09 | 3.67 | -0.14 | 0.01 | - | - | - | - | - | - | - | - | - |
|  | 20 | 574246240 | C/T | 0.02 | - | - | - | - | - | - | 4.68 | -0.45 | 0.02 | - | - | - |
|  | 21 | 43573217 | G/A | 0.02 | - | - | - | 4.42 | 0.56 | 0.03 | - | - | - | - | - | - |
| Floret Shattering | 2 | 333426473 | G/A | 0.41 | - | - | - | - | - | - | 3.99 | -0.26 | 0.05 | - | - | - |
|  | 4 | 183774473 | A/G | 0.16 | - | - | - | 4.63 | -0.43 | 0.03 | - | - | - | - | - | - |
|  | 4 | 247408826 | C/T | 0.03 | 5.27 | -1.04 | 0 | - | - | - | - | - | - | - | - | - |
|  | 5 | 299979105 | A/G | 0.05 | - | - | - | 4.22 | -1.3 | 0.02 | - | - | - | - | - | - |
|  | 5 | 324907771 | T/G | 0.25 | - | - | - | - | - | - | 3.81 | 0.26 | 0.02 | - | - | - |
|  | 5 | 391948474 | C/G | 0.39 | - | - | - | - | - | - | - | - | - | 4.51 | -0.05 | 0.01 |
|  | 5 | 394992438 | G/C | 0.03 | 5.45 | -1.05 | 0.03 | - | - | - | - | - | - | - | - | - |
|  | 6 | 32397489 | C/T | 0.05 | - | - | - | - | - | - | - | - | - | 5.32 | 0.08 | 0.02 |
|  | 7 | 462853440 | G/T | 0.46 | - | - | - | - | - | - | 3.81 | -0.33 | 0.01 | - | - | - |
|  | 7 | 484255571 | C/G | 0.06 | 3.96 | -0.58 | 0.03 | - | - | - | - | - | - | - | - | - |
|  | 8 | 72972971 | T/C | 0.5 | - | - | - | - | - | - | - | - | - | 5.32 | -0.46 | 0.02 |
|  | 9 | 57079262 | C/A | 0.05 | - | - | - | - | - | - | - | - | - | 4.87 | 0.07 | 0.01 |
|  | 10 | 186735495 | T/A | 0.05 | - | - | - | - | - | - | - | - | - | 3.69 | 0.07 | 0 |
|  | 11 | 11700200 | G/C | 0.02 | - | - | - | - | - | - | - | - | - | 3.99 | 0.09 | 0.01 |
|  | 11 | 231841692 | C/T | 0.43 | - | - | - | - | - | - | 9.26 | -0.33 | 0.08 | - | - | - |
|  | 13 | 282529292 | A/T | 0.49 | - | - | - | 5.18 | -0.88 | 0.01 | - | - | - | - | - | - |
|  | 14 | 511253465 | G/T | 0.23 | - | - | - | 5.23 | 0.31 | 0.02 | - | - | - | - | - | - |
|  | 15 | 24540664 | G/A | 0.29 | 3.98 | -0.35 | 0.01 | - | - | - | - | - | - | - | - | - |
|  | 15 | 360964073 | G/A | 0.02 | - | - | - | 4.68 | 0.92 | 0.02 | - | - | - | - | - | - |
|  | 16 | 13393236 | T/A | 0.03 | - | - | - | 3.91 | 0.55 | 0.01 | - | - | - | - | - | - |
|  | 16 | 317104001 | G/A | 0.1 | - | - | - | - | - | - | - | - | - | 3.87 | 0.05 | 0.01 |
|  | 19 | 632334545 | G/A | 0.03 | 3.71 | 0.99 | 0.2 | - | - | - | - | - | - | - | - | - |
|  | 19 | 778538126 | T/C | 0.4 | - | - | - | - | - | - | 5.5 | -0.25 | 0 | - | - | - |
|  | 20 | 78921696 | C/G | 0.08 | 3.8 | 0.48 | 0.02 | - | - | - | - | - | - | - | - | - |
|  | 20 | 199689244 | T/A | 0.01 | - | - | - | - | - | - | - | - | - | 4.84 | 0.16 | 0.02 |
|  | 20 | 593264859 | T/C | 0.09 | - | - | - | 4.69 | -0.37 | 0.02 | - | - | - | - | - | - |
| Threshability | 2 | 394054035 | G/T | 0.02 | - | - | - | - | - | - | - | - | - | 4.64 | -0.09 | 0.03 |
|  | 5 | 86058515 | G/A | 0.35 | - | - | - | - | - | - | 11.39 | -0.09 | 0.04 | - | - | - |
|  | 5 | 156358046 | G/T | 0.35 | - | - | - | - | - | - | 12.72 | -0.1 | 0.08 | - | - | - |
|  | 5 | 232392097 | C/T | 0.39 | - | - | - | - | - | - | 4.08 | -0.05 | 0 | - | - | - |
|  | 5 | 261602958 | T/A | 0.05 | - | - | - | 3.81 | -0.1 | 0.03 | - | - | - | - | - | - |
|  | 5 | 263265303 | G/A | 0.3 | 6.96 | -0.08 | 0.06 | - | - | - | - | - | - | - | - | - |
|  | 6 | 64457302 | C/G | 0.03 | - | - | - | - | - | - | - | - | - | 3.63 | 0.08 | 0.03 |
|  | 7 | 506836505 | T/C | 0.01 | 3.86 | -0.14 | 0.01 | - | - | - | - | - | - | - | - | - |
|  | 8 | 72972971 | T/C | 0.5 | - | - | - | - | - | - | - | - | - | 4.66 | 0.38 | 0.02 |
|  | 11 | 32523201 | G/A | 0.02 | - | - | - | 3.88 | -0.13 | 0.01 | - | - | - | - | - | - |
|  | 13 | 237811341 | A/G | 0.02 | - | - | - | 3.72 | -0.14 | 0 | - | - | - | - | - | - |
|  | 15 | 97902578 | T/C | 0.02 | - | - | - | 3.88 | -0.14 | 0.02 | - | - | - | - | - | - |
|  | 16 | 315495424 | G/C | 0.32 | 3.63 | -0.04 | 0.03 | - | - | - | - | - | - | - | - | - |
|  | 19 | 163945333 | C/T | 0.02 | 3.77 | -0.1 | 0.02 | - | - | - | - | - | - | - | - | - |
|  | 20 | 1840999 | A/G | 0.03 | - | - | - | - | - | - | 4 | -0.11 | 0.02 | - | - | - |

**Table S2.** Homeologous chromosomes in intermediate wheatgrass and their colinear relationship to chromosomes in barley.

| **Intermediate Wheatgrass** | **Homeologous Group** | **Barley** |
| --- | --- | --- |
| 1 | 1 | 1H |
| 2 | 1 | 1H |
| 3 | 1 | 1H |
| 4 | 2 | 2H |
| 5 | 2 | 2H |
| 6 | 2 | 2H |
| 7 | 3 | 3H |
| 8 | 3 | 3H |
| 9 | 3 | 3H |
| 10 | 4 | 4H |
| 11 | 4 | 4H |
| 12 | 4 | 4H |
| 13 | 5 | 5H |
| 14 | 5 | 5H |
| 15 | 5 | 5H |
| 16 | 6 | 6H |
| 17 | 6 | 6H |
| 18 | 6 | 6H |
| 19 | 7 | 7H |
| 20 | 7 | 7H |
| 21 | 7 | 7H |

**Table S3.** Candidate domestication orthologs, their marker name as shown in Figure 4 with the chromosome (Chr) and position (Pos) of significant BLAST hits in intermediate wheatgrass version 2 draft genome (access provided by the Intermediate Wheatgrass Genome Sequencing Consortium) as reported by DeHaan et al., 2020.

| **Candidate Gene** | **Gene ID** | **Trait** | **Source** | **Reference** | **GeneBank ID** | **Version** | **Protein ID** | **E-value** | **Score** | **Identity** | **IWG_V2.0_gene** | **Linkage Group** | **Homeologous Group** | **Physical Position (bp)** |
| --- | --- | --- | --- | --- | --- | --- | --- | --- | --- | --- | --- | --- | --- | --- |
| Brittle rachis | Btr1 | Brittle rachis | barley (3H) | Pourkheirandish et al. 2015 | KX722223 | KX722223.1 | AQP31162.1 | 4.68E-114 | 831.00 | 88.27 | Thintv21093690m.g | 7 | 3 | Chr07:64785511..64786102 |
| Brittle rachis | Btr1 | Brittle rachis | barley (3H) | Pourkheirandish et al. 2015 | KX722223 | KX722223.1 | AQP31162.1 | 9.53E-112 | 815.00 | 86.73 | Thintv21093686m.g | 7 | 3 | Chr07:64714619..64715602 |
| Brittle rachis | Btr1 | Brittle rachis | barley (3H) | Pourkheirandish et al. 2015 | KX722223 | KX722223.1 | AQP31162.1 | 1.61E-107 | 788.00 | 85.64 | Thintv21093684m.g | 7 | 3 | Chr07:64705466..64706908 |
| Brittle rachis | Btr1 | Brittle rachis | barley (3H) | Pourkheirandish et al. 2015 | KX722223 | KX722223.1 | AQP31162.1 | 6.66E-69 | 531 | 66.47 | Thintv21093726m | 7 | 3 | Chr07:66343485..66344288 |
| Brittle rachis | Btr1 | Brittle rachis | barley (3H) | Pourkheirandish et al. 2015 | KX722223 | KX722223.1 | AQP31162.1 | 2.35E-61 | 477 | 90.76 | Thintv21093703m | 7 | 3 | Chr07:65260037..65260668 |
| Brittle rachis | Btr1 | Brittle rachis | barley (3H) | Pourkheirandish et al. 2015 | KX722223 | KX722223.1 | AQP31162.1 | 9.73E-57 | 452 | 56.63 | Thintv21109972m | 8 | 3 | Chr08:37809930..37810508 |
| Brittle rachis | Btr1 | Brittle rachis | barley (3H) | Pourkheirandish et al. 2015 | KX722223 | KX722223.1 | AQP31162.1 | 1.54E-119 | 867.00 | 87.24 | Thintv21125633m.g | 9 | 3 | Chr09:26175732..26176323 |
| Brittle rachis | Btr1 | Brittle rachis | barley (3H) | Pourkheirandish et al. 2015 | KX722223 | KX722223.1 | AQP31162.1 | 9.11E-81 | 612 | 67.35 | Thintv21125614m | 9 | 3 | Chr09:25661113..25668681 |
| Brittle rachis | Btr1 | Brittle rachis | barley (3H) | Pourkheirandish et al. 2015 | KX722223 | KX722223.1 | AQP31162.1 | 2.07E-80 | 609 | 67.35 | Thintv21125628m | 9 | 3 | Chr09:25969063..25972391 |
| Brittle rachis | Btr2 | Brittle rachis | barley (3H) | Pourkheirandish et al. 2015 | KX722226 | KX722226.1 | AQP31165.1 | 2.49E-106 | 781.00 | 81.19 | Thintv21093724m.g | 7 | 3 | Chr07:66141197..66141794 |
| Brittle rachis | Btr2 | Brittle rachis | barley (3H) | Pourkheirandish et al. 2015 | KX722226 | KX722226.1 | AQP31165.1 | 1.76E-111 | 815.00 | 84.65 | Thintv21109969m.g | 8 | 3 | Chr08:37755337..37755934 |
| Brittle rachis | Btr2 | Brittle rachis | barley (3H) | Pourkheirandish et al. 2015 | KX722226 | KX722226.1 | AQP31165.1 | 1.10E-110 | 809.00 | 85.64 | Thintv21125632m.g | 9 | 3 | Chr09:26060998..26061598 |
| naked | Nud | Free threshing | barley (7H) | Yu et al. 2016 | KP245804 | KP245804.1 | AKF40403.1 | 5.29E-91 | 685.00 | 90.14 | Thintv21282433m.g | 19 | 7 | Chr19:510120134..510121796 |
| naked | Nud | Free threshing | barley (7H) | Yu et al. 2016 | KP245804 | KP245804.1 | AKF40403.1 | 6.39E-91 | 685.00 | 87.85 | Thintv21312339m.g | 20 | 7 | Chr20:491744614..491746249 |
| naked | Nud | Free threshing | barley (7H) | Yu et al. 2016 | KP245804 | KP245804.1 | AKF40403.1 | 3.21E-93 | 700.00 | 98.12 | Thintv21331531m.g | 21 | 7 | Chr21:313566208..313567794 |
| spelt factor (free threshing) | Q | Free threshing | wheat (5A) | Simons et al. 2006 | AY702956 | AY702956.1 | AAU94922.1 | 0.00E+00 | 1486.00 | 91.92 | Thintv21187322m.g | 13 | 5 | Chr13:365961283..365964718 |
| spelt factor (free threshing) | Q | Free threshing | wheat (5A) | Simons et al. 2006 | AY702956 | AY702956.1 | AAU94922.1 | 0.00E+00 | 1716.00 | 90.85 | Thintv21202370m.g | 14 | 5 | Chr14:418100272..418103770 |
| QTL of seed shattering in chr1 | qSH1 | Free threshing | rice (1) | Konishi et al. 2006 | AB071330 | AB071330.1 | BAB85942.1 | 4.33E-131 | 1,003.00 | 72 | Thintv21102037m.g | 7 | 3 | Chr07:376399378..376403732 |
| QTL of seed shattering in chr1 | qSH1 | Free threshing | rice (1) | Konishi et al. 2006 | AB071330 | AB071330.1 | BAB85942.1 | 9.53E-128 | 982 | 70.05 | Thintv21102402m.g | 7 | 3 | Chr07:387186179..387190386 |
| QTL of seed shattering in chr1 | qSH1 | Free threshing | rice (1) | Konishi et al. 2006 | AB071330 | AB071330.1 | BAB85942.1 | 1.44E-157 | 1,216.00 | 64.48 | Thintv21135945m.g | 9 | 3 | Chr09:369731903..369737076 |
| shattering QTL chr4 | sh4 | Free threshing | rice (4) | Li et al. 2006 | LOC_Os04g57530 | LOC_Os04g57530.1 | LOC_Os04g57530.1 | 7.96E-33 | 319 | 48.59 | Thintv21140792m | 10 | 4 | Chr10:26172505..26174349 |
| shattering QTL chr4 | sh4 | Free threshing | rice (4) | Li et al. 2006 | LOC_Os04g57530 | LOC_Os04g57530.1 | LOC_Os04g57530.1 | 5.61E-33 | 321 | 48.25 | Thintv21154807m | 11 | 4 | Chr11:8879818..8883404 |
| shattering QTL chr4 | sh4 | Free threshing | rice (4) | Li et al. 2006 | LOC_Os04g57530 | LOC_Os04g57530.1 | LOC_Os04g57530.1 | 3.48E-33 | 321 | 45.77 | Thintv21172232m | 12 | 4 | Chr12:377628308..377629621 |
| SH1-like gene on chr5 | SH5 | Free threshing | rice (5) | Yoon et al. 2014 | Os05 g38120 | LOC_Os05g38120.1 | LOC_Os05g38120.1 | 0.00E+00 | 1,515.00 | 71.3 | Thintv21009159m | 1 | 1 | Chr01:324225555..324229937 |
| SH1-like gene on chr5 | SH5 | Free threshing | rice (5) | Yoon et al. 2014 | Os05 g38120 | LOC_Os05g38120.1 | LOC_Os05g38120.1 | 0.00E+00 | 1,563.00 | 72.12 | Thintv21029605m | 2 | 1 | Chr02:334447331..334451638 |
| SH1-like gene on chr5 | SH5 | Free threshing | rice (5) | Yoon et al. 2014 | Os05 g38120 | LOC_Os05g38120.1 | LOC_Os05g38120.1 | 0.00E+00 | 1,552.00 | 73.55 | Thintv21047090m | 3 | 1 | Chr03:464140241..464144799 |
| Shattering abortion 1 | SHAT1 | Free threshing | rice (4) | Zhou et al. 2012 | LOC_Os04g55560 | LOC_Os04g55560.2 | LOC_Os04g55560.2 | 6.60E-127 | 970.00 | 84.50 | Thintv21058456m.g | 4 | 2 | Chr04:234390930..234394578 |
| Shattering abortion 1 | SHAT1 | Free threshing | rice (4) | Zhou et al. 2012 | LOC_Os04g55560 | LOC_Os04g55560.2 | LOC_Os04g55560.2 | 5.06E-127 | 972.00 | 84.56 | Thintv21074524m.g | 6 | 2 | Chr06:46055017..46059036 |

**Table S4.** Mean phenotypic values for the parents of the intermediate wheatgrass Nested Association Mapping (NAM) population across traits all traits and environments, where STP = St. Paul, MN and TLI = The Land Institute in Salina, KS in 2017 and 2018.

| Brittle Rachis (count) | |  |  |  |
| --- | --- | --- | --- | --- |
| Parent | STP_2017 | STP_2018 | TLI_2017 | TLI_2018 |
| WGN07 | 0.33 | 0 | 0 | 0 |
| WGN15 | 0.4 | 0 | 0.5 | 0 |
| WGN26 | 1 | 0.33 | 0.17 | 0 |
| WGN36 | 0.83 | 1.33 | 0.33 | 0.4 |
| WGN38 | 0.4 | 0.4 | 0.17 | 0.17 |
| WGN39 | 1 | 0.67 | 0.5 | 0 |
| WGN45 | 0 | 0.17 | 0 | 0 |
| WGN46 | 0.6 | 0.75 | 0.33 | 0.5 |
| WGN55 | 0.83 | 0.4 | 0.17 | 0 |
| WGN59 | 1.18 | 1.44 | 1.28 | 0.02 |
| WGN63 | 0.67 | 1 | 1.17 | 0.33 |
|  |  |  |  |  |
| Floret Shattering (scale – see Methods) | |  |  |  |
| Parent | STP_2017 | STP_2018 | TLI_2017 | TLI_2018 |
| WGN07 | 1.67 | 1.83 | 1.5 | 0 |
| WGN15 | 0 | 0 | 0.33 | 0 |
| WGN26 | 4.17 | 4.67 | 3.33 | 0.5 |
| WGN36 | 0.83 | 0.83 | 1.17 | 0 |
| WGN38 | 0.6 | 1.6 | 0.33 | 0 |
| WGN39 | 6.17 | 5.17 | 2.17 | 0.25 |
| WGN45 | 0.4 | 0.83 | 0.83 | 0 |
| WGN46 | 1 | 0.75 | 1 | 0 |
| WGN55 | 2.33 | 0.6 | 2.5 | 0.2 |
| WGN59 | 0.12 | 0.1 | 0.64 | 0 |
| WGN63 | 2.5 | 3.67 | 1.5 | 0 |
|  |  |  |  |  |
| Threshability (% dehulled seed) | |  |  |  |
| Parent | STP_2017 | STP_2018 | TLI_2017 | TLI_2018 |
| WGN07 | 98.33 | 93.67 | 87.33 | 99.75 |
| WGN15 | 93.2 | 83 | 74.17 | 100 |
| WGN26 | 88.17 | 82.5 | 78.33 | 90 |
| WGN36 | 94.67 | 88.17 | 85.83 | 87.5 |
| WGN38 | 82 | 64 | 59.17 | 99.67 |
| WGN39 | 85.33 | 65 | 70 | 100 |
| WGN45 | 91.83 | 93.17 | 84.17 | 91.33 |
| WGN46 | 95.4 | 92 | 75 | 88.33 |
| WGN55 | 92.67 | 89.4 | 81.67 | 99.5 |
| WGN59 | 97.48 | 97.52 | 87.51 | 99.51 |
| WGN63 | 70.83 | 50.83 | 63.33 | 100 |

**Table S5.** Pearson correlation estimates and p-values from analysis of the difference between parental phenotypic values and the standard deviation among their progeny. The analysis was run for three traits in the intermediate wheatgrass Nested Association Mapping (NAM) population: brittle rachis, floret shattering, and threshability across four growing environments (STP = St. Paul, MN; TLI = The Land Institute in Salina, KS in 2017 and 2018). Significance was determined at the $\propto$= 0.05 threshold.

|  | **STP 2017** | | **STP 2018** | | **TLI 2017** | | **TLI 2018** | |
| --- | --- | --- | --- | --- | --- | --- | --- | --- |
| **Trait** | **Estimate** | **p-value** | **Estimate** | **p-value** | **Estimate** | **p-value** | **Estimate** | **p-value** |
| Brittle Rachis | 0.11 | 0.76 | -0.62 | 0.05 | -0.53 | 0.12 | 0.36 | 0.31 |
| Floret Shattering | 0.72 | 0.02 | 0.73 | 0.02 | 0.58 | 0.08 | 0.65 | 0.04 |
| Threshability | 0.57 | 0.09 | 0.75 | 0.01 | 0.55 | 0.1 | -0.25 | 0.49 |

**Table S6.** Results from linkage mapping analyses combined across families and within families for brittle rachis, floret shattering, and threshability, including two-LOD drop off intervals, peak loci, maximum LOD, variance explained and allele effects.

| Trait | Location | Year | Chr^a^ | Map^b^ | Left^c^ | Center^d^ | Right^e^ | Locus^f^ | Max LOD | Analysis^g^ | **r^2^** | **μ^h^** | **α^i^** | **𝛾^j^** |
| --- | --- | --- | --- | --- | --- | --- | --- | --- | --- | --- | --- | --- | --- | --- |
| Floret Shattering | STP | 2017 | 2 | CP | 56.29 | 76.80 | 89.14 | CP_Chr02_343055305 | 7.98 | combined | - | - | - | - |
|  | STP | 2017 | 2 | DP | 76.88 | 84.98 | 97.46 | - | 9.16 | combined | - | - | - | - |
|  | STP | 2017 | 2 | DP | 50.95 | 52.78 | 107.68 | DP_Chr02_9740407 | 3.23 | WGN26 | 8.70 | 1.26 | - | 0.33 |
|  | STP | 2017 | 3 | CP | 25.68 | 30.91 | 48.50 | CP_Chr03_78858449 | 7.95 | combined | - | - | - | - |
|  | STP | 2017 | 4 | DP | 195.52 | 196.54 | 208.54 | - | 14.68 | combined | - | - | - | - |
|  | STP | 2017 | 4 | DP | 195.52 | 201.54 | 201.54 | - | 4.77 | WGN15 | 14.50 | 0.67 | - | 0.36 |
|  | STP | 2017 | 4 | DP | 188.20 | 196.54 | 196.54 | - | 5.47 | WGN55 | 11.90 | 1.52 | - | 0.49 |
|  | STP | 2017 | 5 | CP | 126.22 | 134.96 | 138.26 | - | 18.4 | combined | - | - | - | - |
|  | STP | 2017 | 5 | CP | 95.58 | 128.86 | 146.36 | - | 3.33 | WGN26 | 15.40 | 1.21 | -0.41 | - |
|  | STP | 2017 | 5 | CP | 84.56 | 102.30 | 113.27 | - | 6.47 | WGN55 | 16.50 | 1.46 | -0.53 | - |
|  | STP | 2017 | 5 | DP | 115.78 | 127.77 | 132.79 | - | 8.09 | combined | - | - | - | - |
|  | STP | 2017 | 5 | DP | 101.83 | 128.77 | 132.79 | DP_Chr05_392031395 | 3.73 | WGN15 | 29.60 | 0.52 | - | -1.13 |
|  | STP | 2017 | 5 | DP | 101.83 | 109.76 | 131.28 | - | 4.5 | WGN63 | 12.00 | 1.95 | - | -0.57 |
|  | STP | 2017 | 8 | CP | 101.24 | 108.73 | 116.46 | CP_Chr08_303295574 | 7.8 | combined | - | - | - | - |
|  | STP | 2017 | 11 | CP | 106.79 | 109.19 | 111.34 | - | 31.98 | combined | - | - | - | - |
|  | STP | 2017 | 11 | CP | 101.44 | 109.40 | 121.29 | CP_Chr11_238097236 | 4.64 | WGN26 | 15.10 | 1.21 | -0.41 | - |
|  | STP | 2017 | 11 | CP | 101.44 | 106.79 | 112.34 | CP_Chr11_236555758 | 4.75 | WGN39 | 14.80 | 3.05 | -0.66 | - |
|  | STP | 2017 | 11 | CP | 93.62 | 97.60 | 111.34 | - | 5.74 | WGN45 | 23.70 | 1.12 | -0.49 | - |
|  | STP | 2017 | 11 | CP | 102.44 | 115.29 | 122.29 | CP_Chr11_231841692 | 3.62 | WGN46 | 12.90 | 1.17 | -0.46 | - |
|  | STP | 2017 | 11 | CP | 87.39 | 103.44 | 115.26 | - | 5.7 | WGN55 | 15.20 | 1.49 | -0.49 | - |
|  | STP | 2017 | 17 | CP | 22.28 | 41.85 | 54.18 | - | 4.06 | WGN45 | 28.90 | 1.06 | -0.43 | - |
|  | STP | 2018 | 2 | CP | 86.14 | 103.61 | 130.56 | - | 3.77 | WGN39 | 12.50 | 2.54 | 0.52 | - |
|  | STP | 2018 | 2 | DP | 75.88 | 91.41 | 112.43 | - | 8.65 | combined | - | - | - | - |
|  | STP | 2018 | 2 | DP | 70.59 | 79.84 | 79.84 | DP_Chr02_338749432 | 3.57 | WGN38 | 10.60 | 0.71 | - | -0.24 |
|  | STP | 2018 | 4 | DP | 182.20 | 195.54 | 195.54 | DP_Chr04_260663237 | 12.21 | combined | - | - | - | - |
|  | STP | 2018 | 4 | DP | 177.20 | 187.20 | 187.20 | - | 5.42 | WGN55 | 28.20 | 1.25 | - | 0.42 |
|  | STP | 2018 | 5 | CP | 128.86 | 135.96 | 146.36 | - | 16.03 | combined | - | - | - | - |
|  | STP | 2018 | 5 | CP | 6.11 | 63.00 | 83.55 | CP_Chr05_86148562 | 3.18 | WGN26 | 9.20 | 1.12 | 0.33 | - |
|  | STP | 2018 | 5 | CP | 116.92 | 135.96 | 146.36 | - | 4.47 | WGN38 | 16.20 | 0.67 | 0.29 | - |
|  | STP | 2018 | 5 | CP | 113.27 | 118.23 | 146.36 | - | 5.26 | WGN55 | 13.80 | 1.24 | -0.33 | - |
|  | STP | 2018 | 5 | DP | 143.36 | 146.36 | 146.36 | DP_Chr05_339720810 | 9.36 | combined | - | - | - | - |
|  | STP | 2018 | 5 | DP | 143.36 | 146.36 | 146.36 | DP_Chr05_339720810 | 7.37 | WGN07 | 41.00 | 0.72 | - | 1.78 |
|  | STP | 2018 | 8 | CP | 108.93 | 120.51 | 153.63 | CP_Chr08_317239119 | 3.77 | WGN45 | 8.90 | 1.12 | 0.30 | - |
|  | STP | 2018 | 9 | CP | 122.49 | 131.89 | 137.77 | CP_Chr09_111456911 | 11.25 | combined | - | - | - | - |
|  | STP | 2018 | 9 | CP | 93.31 | 130.41 | 140.17 | CP_Chr09_447902332 | 3.35 | WGN26 | 11.50 | 1.15 | 0.35 | - |
|  | STP | 2018 | 11 | CP | 107.79 | 109.19 | 111.34 | - | 35.04 | combined | - | - | - | - |
|  | STP | 2018 | 11 | CP | 101.44 | 108.19 | 112.34 | CP_Chr11_244241276 | 4.53 | WGN07 | 15.40 | 0.87 | -0.39 | - |
|  | STP | 2018 | 11 | CP | 80.64 | 101.44 | 115.26 | CP_Chr11_228740879 | 5.38 | WGN15 | 14.90 | 0.66 | -0.31 | - |
|  | STP | 2018 | 11 | CP | 96.60 | 109.19 | 109.19 | - | 3.37 | WGN26 | 11.10 | 1.15 | -0.36 | - |
|  | STP | 2018 | 11 | CP | 106.79 | 109.40 | 109.40 | CP_Chr11_238097236 | 5.53 | WGN38 | 19.80 | 0.68 | 0.32 | - |
|  | STP | 2018 | 11 | CP | 90.84 | 104.44 | 122.29 | - | 4.32 | WGN39 | 15.40 | 2.44 | -0.55 | - |
|  | STP | 2018 | 11 | CP | 106.79 | 122.29 | 122.29 | - | 6.43 | WGN45 | 27.20 | 1.09 | -0.48 | - |
|  | STP | 2018 | 11 | CP | 80.64 | 91.81 | 106.79 | CP_Chr11_225969790 | 5.57 | WGN55 | 12.40 | 1.21 | -0.31 | - |
|  | TLI | 2017 | 2 | CP | 55.29 | 77.54 | 86.14 | CP_Chr02_338749407 | 12.37 | combined | - | - | - | - |
|  | TLI | 2017 | 2 | CP | 41.29 | 76.80 | 102.61 | CP_Chr02_343055305 | 3.57 | WGN38 | 14.60 | 1.14 | 0.38 | - |
|  | TLI | 2017 | 2 | DP | 66.33 | 77.88 | 109.68 | - | 9.21 | combined | - | - | - | - |
|  | TLI | 2017 | 5 | CP | 129.86 | 135.96 | 146.36 | - | 16.89 | combined | - | - | - | - |
|  | TLI | 2017 | 5 | CP | 126.86 | 134.96 | 146.36 | - | 4.3 | WGN45 | 15.30 | 1.15 | -0.31 | - |
|  | TLI | 2017 | 8 | CP | 106.73 | 119.11 | 130.73 | CP_Chr08_317351181 | 3.5 | WGN45 | 10.50 | 1.15 | 0.26 | - |
|  | TLI | 2017 | 11 | CP | 104.44 | 109.19 | 117.29 | - | 21.73 | combined | - | - | - | - |
|  | TLI | 2017 | 11 | CP | 85.57 | 109.19 | 109.19 | - | 4.21 | WGN36 | 15.40 | 1.11 | -0.36 | - |
|  | TLI | 2017 | 11 | CP | 94.60 | 113.33 | 113.33 | CP_Chr11_234190857 | 4.47 | WGN45 | 15.80 | 1.13 | -0.32 | - |
|  | TLI | 2017 | 19 | CP | 112.76 | 129.50 | 141.72 | CP_Chr19_561090027 | 3.98 | WGN38 | 12.40 | 1.11 | 0.33 | - |
|  | TLI | 2018 | 15 | DP | 74.93 | 124.04 | 124.04 | - | 3.32 | WGN46 | 12.20 | 0.07 | - | 0.06 |
| Brittle Rachis | STP | 2017 | 4 | DP | 80.58 | 83.89 | 113.64 | DP_Chr04_220417527 | 9.42 | combined | - | - | - | - |
|  | STP | 2017 | 5 | DP | 55.56 | 83.07 | 114.76 | - | 17.3 | combined | - | - | - | - |
|  | STP | 2017 | 5 | DP | 55.56 | 82.07 | 109.76 | - | 15.61 | WGN07 | 33.60 | 0.52 | - | 0.26 |
|  | STP | 2017 | 6 | CP | 161.04 | 163.14 | 163.14 | CP_Chr06_529546708 | 15.32 | combined | - | - | - | - |
|  | STP | 2017 | 6 | CP | 161.04 | 163.14 | 163.14 | CP_Chr06_529546708 | 12.11 | WGN07 | 33.60 | 0.52 | -0.26 | - |
|  | STP | 2017 | 8 | DP | 55.46 | 82.02 | 89.60 | - | 8.14 | combined | - | - | - | - |
|  | STP | 2017 | 19 | DP | 0.00 | 32.57 | 45.68 | - | 17.94 | combined | - | - | - | - |
|  | STP | 2017 | 19 | DP | 0.00 | 0.00 | 45.68 | DP_Chr19_96269829 | 15.63 | WGN07 | 33.60 | 0.52 | - | 0.26 |
|  | STP | 2018 | 1 | CP | 124.08 | 126.64 | 133.41 | CP_Chr01_410726049 | 12.71 | combined | - | - | - | - |
|  | STP | 2018 | 8 | CP | 61.03 | 68.67 | 74.83 | CP_Chr08_23347234 | 9.73 | combined | - | - | - | - |
|  | STP | 2018 | 8 | DP | 64.06 | 82.02 | 89.60 | - | 11.08 | combined | - | - | - | - |
|  | STP | 2018 | 8 | DP | 58.46 | 82.02 | 89.60 | - | 6.26 | WGN45 | 23.30 | 0.62 | - | 0.33 |
|  | TLI | 2017 | 8 | CP | 64.74 | 75.63 | 87.38 | CP_Chr08_22429423 | 11.85 | combined | - | - | - | - |
|  | TLI | 2017 | 8 | CP | 64.74 | 76.98 | 106.30 | - | 3.6 | WGN36 | 14.10 | 0.58 | 0.23 | - |
|  | TLI | 2017 | 9 | DP | 25.32 | 63.61 | 81.95 | DP_Chr09_300947779 | 3.16 | WGN26 | 12.20 | 0.58 | - | -0.20 |
| Threshability | STP | 2017 | 5 | CP | 64.15 | 65.78 | 66.61 | CP_Chr05_184445451 | 45.77 | combined | - | - | - | - |
|  | STP | 2017 | 5 | CP | 2.11 | 65.94 | 71.90 | CP_Chr05_219902957 | 5.92 | WGN15 | 20.00 | 1.24 | -0.10 | - |
|  | STP | 2017 | 5 | CP | 54.69 | 65.47 | 78.23 | CP_Chr05_111147787 | 10.79 | WGN26 | 28.00 | 1.29 | -0.11 | - |
|  | STP | 2017 | 5 | CP | 23.50 | 65.47 | 84.55 | CP_Chr05_111147787 | 4.57 | WGN36 | 16.60 | 1.34 | 0.06 | - |
|  | STP | 2017 | 5 | CP | 41.97 | 60.66 | 77.23 | - | 10.02 | WGN38 | 36.30 | 1.29 | 0.12 | - |
|  | STP | 2017 | 5 | CP | 63.00 | 65.47 | 66.61 | CP_Chr05_111147787 | 10.35 | WGN63 | 34.00 | 1.16 | -0.10 | - |
|  | STP | 2017 | 5 | DP | 42.98 | 62.79 | 69.46 | DP_Chr05_196581222 | 10.81 | combined | - | - | - | - |
|  | STP | 2017 | 5 | DP | 40.97 | 64.13 | 69.46 | - | 7.87 | WGN15 | 21.20 | 1.24 | - | 0.10 |
|  | STP | 2017 | 11 | DP | 67.29 | 91.29 | 91.29 | - | 3.54 | WGN39 | 13.50 | 1.21 | - | -0.06 |
|  | STP | 2017 | 18 | DP | 71.56 | 74.80 | 144.53 | DP_Chr18_206116238 | 3.41 | WGN15 | 9.50 | 1.24 | - | -0.07 |
|  | STP | 2017 | 19 | DP | 60.31 | 88.89 | 109.97 | DP_Chr19_131389206 | 8.84 | combined | - | - | - | - |
|  | STP | 2017 | 20 | DP | 19.15 | 40.80 | 73.05 | - | 3.54 | WGN15 | 17.20 | 1.24 | - | -0.09 |
|  | STP | 2017 | 21 | CP | 114.23 | 126.57 | 126.57 | CP_Chr21_395786372 | 3.55 | WGN55 | 13.70 | 1.36 | 0.03 | - |
|  | STP | 2018 | 3 | DP | 44.61 | 64.55 | 76.95 | DP_Chr03_287042026 | 11.18 | combined | - | - | - | - |
|  | STP | 2018 | 3 | DP | 16.72 | 64.55 | 83.30 | DP_Chr03_287042026 | 4.73 | WGN45 | 16.40 | 1.26 | - | 0.05 |
|  | STP | 2018 | 5 | CP | 63.00 | 65.47 | 66.61 | CP_Chr05_111147787 | 47.04 | combined | - | - | - | - |
|  | STP | 2018 | 5 | CP | 37.97 | 65.47 | 74.02 | CP_Chr05_111147787 | 5.49 | WGN15 | 19.20 | 1.17 | -0.11 | - |
|  | STP | 2018 | 5 | CP | 56.85 | 63.00 | 74.02 | CP_Chr05_86148562 | 12.33 | WGN26 | 36.60 | 1.22 | -0.14 | - |
|  | STP | 2018 | 5 | CP | 57.85 | 57.85 | 89.58 | - | 3.87 | WGN36 | 15.30 | 1.25 | 0.07 | - |
|  | STP | 2018 | 5 | CP | 56.85 | 65.47 | 76.02 | CP_Chr05_111147787 | 13.52 | WGN38 | 48.40 | 1.16 | 0.19 | - |
|  | STP | 2018 | 5 | CP | 51.69 | 65.47 | 76.02 | CP_Chr05_111147787 | 4.07 | WGN39 | 14.20 | 1.07 | 0.08 | - |
|  | STP | 2018 | 5 | CP | 66.61 | 67.87 | 75.02 | CP_Chr05_232392097 | 7.52 | WGN63 | 25.50 | 1.07 | -0.11 | - |
|  | STP | 2018 | 5 | DP | 48.98 | 62.79 | 69.46 | DP_Chr05_196581222 | 10.51 | combined | - | - | - | - |
|  | STP | 2018 | 5 | DP | 47.98 | 64.13 | 81.07 | - | 7.62 | WGN15 | 21.50 | 1.17 | - | 0.11 |
|  | STP | 2018 | 11 | CP | 43.00 | 46.82 | 53.35 | CP_Chr11_34860853 | 10.77 | combined | - | - | - | - |
|  | STP | 2018 | 11 | CP | 37.10 | 59.87 | 73.83 | - | 3.42 | WGN45 | 14.10 | 1.26 | 0.05 | - |
|  | STP | 2018 | 17 | CP | 63.53 | 69.40 | 74.27 | CP_Chr17_228138417 | 8.45 | combined | - | - | - | - |
|  | STP | 2018 | 20 | CP | 79.14 | 85.11 | 90.20 | CP_Chr20_437898043 | 8.09 | combined | - | - | - | - |
|  | TLI | 2017 | 3 | DP | 35.03 | 35.03 | 83.30 | - | 3.5 | WGN45 | 34.50 | 1.10 | - | 0.09 |
|  | TLI | 2017 | 5 | CP | 64.15 | 65.78 | 68.04 | CP_Chr05_184445451 | 60.18 | combined | - | - | - | - |
|  | TLI | 2017 | 5 | CP | 62.59 | 65.94 | 70.44 | CP_Chr05_219902957 | 7.5 | WGN15 | 25.60 | 0.97 | -0.13 | - |
|  | TLI | 2017 | 5 | CP | 58.85 | 70.44 | 81.55 | CP_Chr05_247578147 | 16.83 | WGN26 | 60.80 | 1.09 | -0.15 | - |
|  | TLI | 2017 | 5 | CP | 7.11 | 70.44 | 90.58 | CP_Chr05_247578147 | 5.45 | WGN36 | 24.50 | 1.14 | 0.07 | - |
|  | TLI | 2017 | 5 | CP | 56.85 | 60.66 | 71.90 | - | 16.84 | WGN38 | 58.90 | 1.04 | 0.19 | - |
|  | TLI | 2017 | 5 | CP | 50.69 | 64.48 | 71.90 | CP_Chr05_86058515 | 3.86 | WGN39 | 13.60 | 1.01 | 0.06 | - |
|  | TLI | 2017 | 5 | CP | 56.85 | 59.66 | 71.90 | - | 5.6 | WGN45 | 23.00 | 1.11 | -0.07 | - |
|  | TLI | 2017 | 5 | CP | 63.00 | 68.56 | 75.02 | CP_Chr05_210699121 | 6.53 | WGN63 | 23.90 | 1.02 | -0.07 | - |
|  | TLI | 2017 | 5 | DP | 44.98 | 67.93 | 80.07 | - | 5.53 | WGN15 | 15.30 | 0.97 | - | 0.10 |
|  | TLI | 2017 | 11 | CP | 44.00 | 51.35 | 55.72 | - | 12.06 | combined | - | - | - | - |
|  | TLI | 2017 | 11 | CP | 65.21 | 96.60 | 96.60 | - | 4.53 | WGN55 | 16.90 | 1.16 | 0.05 | - |
|  | TLI | 2017 | 14 | DP | 42.47 | 65.00 | 80.21 | DP_Chr14_136180807 | 3.83 | WGN63 | 11.30 | 1.03 | - | -0.04 |
|  | TLI | 2017 | 19 | CP | 74.72 | 100.94 | 105.74 | CP_Chr19_157334685 | 4.82 | WGN15 | 12.80 | 0.96 | -0.09 | - |
|  | TLI | 2017 | 20 | DP | 21.05 | 47.79 | 75.58 | DP_Chr20_204585011 | 4.3 | WGN15 | 23.90 | 0.97 | - | -0.12 |
|  | TLI | 2017 | 20 | DP | 143.51 | 156.77 | 167.76 | DP_Chr20_590832266 | 3.51 | WGN45 | 11.60 | 1.10 | - | 0.05 |
|  | TLI | 2018 | 6 | DP | 45.54 | 76.80 | 137.29 | DP_Chr06_300299340 | 2.8 | WGN55 | 11.60 | 1.50 | - | -0.06 |
|  | TLI | 2018 | 11 | CP | 0.00 | 101.44 | 125.80 | CP_Chr11_228740879 | 2.05 | WGN45 | 8.90 | 1.52 | 0.04 | - |
|  | TLI | 2018 | 13 | DP | 0.00 | 107.45 | 171.27 | DP_Chr13_252462690 | 1.84 | WGN39 | 8.60 | 1.51 | - | 0.05 |
|  | TLI | 2018 | 21 | DP | 8.22 | 57.00 | 82.38 | DP_Chr21_101931494 | 3.6 | WGN38 | 17.40 | 1.50 | - | 0.05 |

^a^ Chr, chromosome

^b^ Map used in linkage mapping

^c-e^ Genetic positions in centimorgans (cM) of the 2-LOD drop-off interval

^f^ Peak locus of the QTL interval where “-“ indicates that the peak was between two markers.

^g^ Analysis whether the QTL was detected in the combined (including all families) analysis or within the analysis of a specific family. Allele effects are not reported in the combined analysis

^h^ Overall mean

^i^ Difference between alleles in the common parent

^j^ Difference between alleles in the donor parent

**Table S7.** Results from analysis of variance for mixed effects linear models within each environment for each trait. Brittle rachis and floret shattering were analyzed as generalized mixed effects linear models.

| **Brittle Rachis** |  |  |  |  |  |
| --- | --- | --- | --- | --- | --- |
| STP17 |  |  |  |  |  |
|  | Df | Deviance | Resid. Df | Resid. Dev |  |
| NULL | NA | NA | 2295 | 743.6232578 |  |
| famID | 9 | 22.1642563 | 2286 | 721.4590015 |  |
| rep | 1 | 0.244736248 | 2285 | 721.2142653 |  |
| famID:plantID3 | 1173 | 444.9519113 | 1112 | 276.262354 |  |
|  |  |  |  |  |  |
| STP18 |  |  |  |  |  |
|  | Df | Deviance | Resid. Df | Resid. Dev |  |
| NULL | NA | NA | 2320 | 1466.282637 |  |
| famID | 9 | 93.99726841 | 2311 | 1372.285368 |  |
| rep | 1 | 0.847723795 | 2310 | 1371.437645 |  |
| famID:plantID3 | 1187 | 878.9518795 | 1123 | 492.4857651 |  |
|  |  |  |  |  |  |
| TLI17 |  |  |  |  |  |
|  | Df | Deviance | Resid. Df | Resid. Dev |  |
| NULL | NA | NA | 2316 | 1110.558481 |  |
| famID | 9 | 42.27058207 | 2307 | 1068.287899 |  |
| rep | 1 | 0.1178156 | 2306 | 1068.170083 |  |
| famID:plantID3 | 1191 | 673.7708896 | 1115 | 394.3991935 |  |
|  |  |  |  |  |  |
| TLI18 |  |  |  |  |  |
|  | Df | Deviance | Resid. Df | Resid. Dev |  |
| NULL | NA | NA | 2153 | 146.1039926 |  |
| famID | 9 | 4.636114303 | 2144 | 141.4678783 |  |
| rep | 1 | 1.951461149 | 2143 | 139.5164171 |  |
| famID:plantID3 | 1182 | 80.78045038 | 961 | 58.73596674 |  |
|  |  |  |  |  |  |
| **Floret Shattering** |  |  |  |  |  |
| STP17 |  |  |  |  |  |
|  | Df | Deviance | Resid. Df | Resid. Dev |  |
| NULL | NA | NA | 2282 | 5399.611914 |  |
| famID | 9 | 1273.719353 | 2273 | 4125.892561 |  |
| rep | 1 | 6.950632003 | 2272 | 4118.941929 |  |
| famID:plantID3 | 1169 | 3290.653885 | 1103 | 828.2880435 |  |
|  |  |  |  |  |  |
| STP18 |  |  |  |  |  |
|  | Df | Deviance | Resid. Df | Resid. Dev |  |
| NULL | NA | NA | 2317 | 3790.089733 |  |
| famID | 9 | 695.141301 | 2308 | 3094.948432 |  |
| rep | 1 | 0.572251183 | 2307 | 3094.37618 |  |
| famID:plantID3 | 1186 | 2347.250958 | 1121 | 747.1252228 |  |
|  |  |  |  |  |  |
| TLI17 |  |  |  |  |  |
|  | Df | Deviance | Resid. Df | Resid. Dev |  |
| NULL | NA | NA | 2312 | 2782.161695 |  |
| famID | 9 | 309.3255753 | 2303 | 2472.836119 |  |
| rep | 1 | 1.508487866 | 2302 | 2471.327632 |  |
| famID:plantID3 | 1190 | 1782.362672 | 1112 | 688.9649596 |  |
|  |  |  |  |  |  |
| TLI18 |  |  |  |  |  |
|  | Df | Deviance | Resid. Df | Resid. Dev |  |
| NULL | NA | NA | 2139 | 69.02570093 |  |
| famID | 9 | 1.082800196 | 2130 | 67.94290074 |  |
| rep | 1 | 0.7986702 | 2129 | 67.14423054 |  |
| famID:plantID3 | 1180 | 36.90423054 | 949 | 30.24 |  |
|  |  |  |  |  |  |
| **Threshability** |  |  |  |  |  |
| STP17 |  |  |  |  |  |
|  | Df | Sum Sq | Mean Sq | F value | Pr(>F) |
| famID | 9 | 13.83332952 | 1.537036613 | 311.1732516 | 1.85E-296 |
| rep | 1 | 0.073205947 | 0.073205947 | 14.82055297 | 0.000124978 |
| famID:plantID3 | 1173 | 53.14435748 | 0.045306358 | 9.172277681 | 1.23E-255 |
| Residuals | 1112 | 5.492710908 | 0.004939488 | NA | NA |
|  |  |  |  |  |  |
| STP18 |  |  |  |  |  |
|  | Df | Sum Sq | Mean Sq | F value | Pr(>F) |
| famID | 9 | 22.02249133 | 2.446943481 | 231.8747694 | 7.79E-249 |
| rep | 1 | 0.377023775 | 0.377023775 | 35.72714353 | 3.05E-09 |
| famID:plantID3 | 1187 | 82.60222787 | 0.069589072 | 6.594328815 | 1.29E-195 |
| Residuals | 1122 | 11.84031619 | 0.010552866 | NA | NA |
|  |  |  |  |  |  |
| TLI17 |  |  |  |  |  |
|  | Df | Sum Sq | Mean Sq | F value | Pr(>F) |
| famID | 9 | 11.87042417 | 1.318936019 | 220.9186009 | 1.34E-240 |
| rep | 1 | 0.003065705 | 0.003065705 | 0.513498265 | 0.473778591 |
| famID:plantID3 | 1191 | 67.32141033 | 0.056525114 | 9.467820149 | 5.46E-263 |
| Residuals | 1114 | 6.650842072 | 0.005970235 | NA | NA |
|  |  |  |  |  |  |
| TLI18 |  |  |  |  |  |
|  | Df | Sum Sq | Mean Sq | F value | Pr(>F) |
| famID | 9 | 0.353284039 | 0.039253782 | 3.404117504 | 0.000426658 |
| rep | 1 | 0.046102671 | 0.046102671 | 3.998058266 | 0.045989308 |
| famID:plantID3 | 1032 | 21.86420851 | 0.021186249 | 1.837287406 | 1.32E-16 |
| Residuals | 619 | 7.137853237 | 0.011531265 | NA | NA |

**Table S8.** Minimum, mean, maximum and standard deviation for phenotypic values within environments where STP = St. Paul, MN and TLI = The Land Institute in Salina, KS in 2017 and 2018 for each trait.

|  | **STP 2017** | | | | **STP 2018** | | | | **TLI 2017** | | | | **TLI 2018** | | | |
| --- | --- | --- | --- | --- | --- | --- | --- | --- | --- | --- | --- | --- | --- | --- | --- | --- |
| **Trait** | **min** | **max** | **mean** | **sd** | **min** | **max** | **mean** | **sd** | **min** | **max** | **mean** | **sd** | **min** | **max** | **mean** | **sd** |
| Brittle Rachis (ct) | -0.01 | 2.5 | 0.8 | 0.46 | -0.02 | 3.5 | 0.78 | 0.65 | -0.01 | 3.5 | 0.58 | 0.56 | -0.03 | 1.5 | 0.06 | 0.2 |
| Floret Shattering (scale) | -0.05 | 7 | 1.26 | 1.42 | -0.01 | 7.99 | 1.16 | 1.16 | -0.02 | 4.02 | 1.36 | 0.96 | -0.02 | 1.98 | 0.03 | 0.14 |
| Threshability (%) | 17.43 | 100 | 91.05 | 11.08 | 7.3 | 100 | 85.63 | 15.81 | 8.97 | 98.97 | 77.03 | 15.39 | 10.34 | 100 | 98.49 | 6.94 |

**Table S9.** Mean of progeny derived from the common parent (C) or the donor parent (D) as the mother within families of the intermediate wheatgrass nested association mapping population across environments for each trait. P-value is the result from a t-test with unequal variance between the two progeny types.

| Brittle Rachis (count) |  |  |  |  |  |  |  |  |  |  |  |  |
| --- | --- | --- | --- | --- | --- | --- | --- | --- | --- | --- | --- | --- |
| Family | C | D | p-value | C | D | p-value | C | D | p-value | C | D | p-value |
| WGN07 | 0.57 | 0.63 | 0.47 | 0.43 | 0.5 | 0.46 | 0.27 | 0.27 | 1 | 0 | 0 | 0.59 |
| WGN15 | 0.83 | 0.8 | 0.59 | 0.61 | 0.68 | 0.49 | 0.67 | 0.68 | 0.91 | 0.03 | 0.02 | 0.76 |
| WGN26 | 0.71 | 0.66 | 0.56 | 0.57 | 0.62 | 0.66 | 0.53 | 0.57 | 0.69 | 0.05 | 0.08 | 0.36 |
| WGN36 | 0.89 | 0.98 | 0.29 | 1.24 | 1.2 | 0.77 | 0.71 | 0.52 | 0.1 | 0.12 | 0.09 | 0.58 |
| WGN38 | 0.87 | 0.92 | 0.64 | 0.96 | 0.95 | 0.97 | 0.48 | 0.5 | 0.82 | 0 | 0.04 | 0.17 |
| WGN39 | 0.91 | 0.87 | 0.63 | 0.95 | 0.78 | 0.14 | 0.74 | 0.61 | 0.26 | 0.14 | 0.15 | 0.91 |
| WGN45 | 0.74 | 0.68 | 0.56 | 0.76 | 0.6 | 0.2 | 0.58 | 0.62 | 0.76 | 0.08 | 0.04 | 0.32 |
| WGN46 | 0.86 | 0.73 | 0.11 | 0.77 | 0.77 | 0.99 | 0.53 | 0.57 | 0.67 | 0.07 | 0.09 | 0.54 |
| WGN55 | 0.8 | 0.76 | 0.58 | 0.86 | 0.73 | 0.18 | 0.61 | 0.48 | 0.19 | 0.03 | 0.02 | 0.76 |
| WGN63 | 0.79 | 0.94 | 0.06 | 0.75 | 1.02 | 0.05 | 0.69 | 0.9 | 0.06 | 0.06 | 0.15 | 0.05 |
|  |  |  |  |  |  |  |  |  |  |  |  |  |
| Floret Shattering (Scale – see Methods) |  |  |  |  |  |  |  |  |  |  |  |  |
| Family | C | D | p-value | C | D | p-value | C | D | p-value | C | D | p-value |
| WGN07 | 0.83 | 0.87 | 0.83 | 0.75 | 0.88 | 0.49 | 1.19 | 1.24 | 0.74 | 0 | 0.01 | 0.12 |
| WGN15 | 0.66 | 0.54 | 0.47 | 0.71 | 0.61 | 0.48 | 0.91 | 0.81 | 0.49 | 0 | 0 | 0.82 |
| WGN26 | 1.05 | 1.13 | 0.69 | 1.21 | 0.92 | 0.15 | 1.75 | 1.7 | 0.79 | 0.06 | 0.01 | 0.27 |
| WGN36 | 0.69 | 0.57 | 0.54 | 0.74 | 0.69 | 0.75 | 1.09 | 1.09 | 0.98 | 0.01 | 0.04 | 0.11 |
| WGN38 | 0.58 | 0.76 | 0.33 | 0.59 | 0.64 | 0.74 | 0.96 | 1.08 | 0.57 | 0.01 | 0.03 | 0.44 |
| WGN39 | 2.79 | 3.33 | 0.07 | 2.31 | 2.53 | 0.37 | 1.94 | 2.15 | 0.18 | 0.02 | 0.05 | 0.4 |
| WGN45 | 0.87 | 0.98 | 0.59 | 0.94 | 0.98 | 0.8 | 1.04 | 1 | 0.8 | 0.01 | 0.01 | 0.69 |
| WGN46 | 1.09 | 1.11 | 0.94 | 1.18 | 1.11 | 0.74 | 1.32 | 1.36 | 0.81 | 0.04 | 0.08 | 0.29 |
| WGN55 | 1.37 | 1.67 | 0.2 | 1.2 | 1.29 | 0.61 | 1.5 | 1.85 | 0.04 | 0.06 | 0.08 | 0.6 |
| WGN63 | 1.84 | 1.95 | 0.68 | 1.97 | 1.62 | 0.14 | 1.54 | 1.46 | 0.57 | 0.03 | 0.01 | 0.39 |
|  |  |  |  |  |  |  |  |  |  |  |  |  |
| Threshability (% clean seed) |  |  |  |  |  |  |  |  |  |  |  |  |
| Family | C | D | p-value | C | D | p-value | C | D | p-value | C | D | p-value |
| WGN07 | 97.14 | 97.48 | 0.51 | 94.93 | 96.04 | 0.21 | 85.04 | 87.97 | 0.05 | 99.77 | 98.84 | 0.28 |
| WGN15 | 87.22 | 85.63 | 0.58 | 83.24 | 79.63 | 0.32 | 68.69 | 65.45 | 0.42 | 97.04 | 99.39 | 0.26 |
| WGN26 | 88.58 | 91.19 | 0.25 | 82.85 | 86.5 | 0.22 | 72.65 | 80.6 | 0.01 | 97.51 | 99.23 | 0.13 |
| WGN36 | 93.88 | 93.48 | 0.79 | 88.75 | 88.09 | 0.77 | 81.93 | 81.32 | 0.78 | 98.78 | 99.44 | 0.47 |
| WGN38 | 89.74 | 87.12 | 0.39 | 81.44 | 76.19 | 0.27 | 71.03 | 69.96 | 0.82 | 98.5 | 99 | 0.48 |
| WGN39 | 87.06 | 85.1 | 0.38 | 77.41 | 73.59 | 0.25 | 71.75 | 71.32 | 0.86 | 97.95 | 98.17 | 0.91 |
| WGN45 | 93.04 | 93.25 | 0.88 | 90.03 | 90.66 | 0.68 | 77.46 | 78.63 | 0.61 | 97.01 | 99.21 | 0.21 |
| WGN46 | 95.03 | 97 | 0.02 | 91.15 | 93.05 | 0.17 | 81.45 | 82.05 | 0.75 | 98.69 | 98.12 | 0.43 |
| WGN55 | 95.99 | 95.32 | 0.14 | 92.82 | 90.73 | 0.03 | 84.56 | 81.28 | 0.02 | 98.43 | 96.5 | 0.31 |
| WGN63 | 83.74 | 83.05 | 0.76 | 75.67 | 76.97 | 0.68 | 73.31 | 72.43 | 0.68 | 99.28 | 99.31 | 0.93 |

**Figure S1.** Shattering drop test method. Three intermediate wheatgrass spikes were dropped horizontally from 20 cm height three times onto a foam tray and disarticulation events were characterized, counted and recorded.

**Figure S2.** Manhattan plots for genome-wide association mapping of brittle rachis type shattering across four environments (STP = St. Paul, MN; TLI = The Land Institute in Salina, KS). Horizontal gray dotted line indicates significance threshold of LOD = 3.6.

**Figure S3.** Manhattan plots for genome-wide association mapping of floret shattering across four environments (STP = St. Paul, MN; TLI = The Land Institute in Salina, KS). Horizontal gray dotted line indicates significance threshold of LOD = 3.6.

**Figure S4**. Manhattan plots for genome-wide association mapping of threshability across four environments (STP = St. Paul, MN; TLI = The Land Institute in Salina, KS). Horizontal gray dotted line indicates significance threshold of LOD = 3.6.

**Additional References not in the Paper:**

Civáň, P., and T.A. Brown. 2017. A novel mutation conferring the nonbrittle phenotype of cultivated barley. New Phytol. 214(1): 468–472. doi: 10.1111/nph.14377.

Konishi, S., T. Izawa, S.Y. Lin, K. Ebana, Y. Fukuta, et al. 2006. An SNP caused loss of seed shattering during rice domestication. Science (80-. ). 312(5778): 1392–1396. doi: 10.1126/science.1126410.

Yu, S., H. Long, G. Deng, Z. Pan, J. Liang, et al. 2016. A Single Nucleotide Polymorphism of Nud Converts the Caryopsis Type of Barley (Hordeum Vulgare L.). Plant Mol. Biol. Report. 34(1): 242–248. doi: 10.1007/s11105-015-0911-9.
